# Phase Ib study of combinations of avadomide (CC‐122), CC‐223, CC‐292, and rituximab in patients with relapsed/refractory diffuse large B‐cell lymphoma

**DOI:** 10.1002/jha2.375

**Published:** 2022-01-14

**Authors:** Vincent Ribrag, Julio C. Chavez, Carola Boccomini, Jason Kaplan, Jason C. Chandler, Armando Santoro, Paolo Corradini, Ian W. Flinn, Ranjana Advani, Philippe A. Cassier, Randeep Sangha, Vaishalee P. Kenkre, Iris Isufi, Shailaja Uttamsingh, Patrick R. Hagner, Anita K. Gandhi, Frank Shen, Sophie Michelliza, Harald Haeske, Kristen Hege, Michael Pourdehnad, John Kuruvilla

**Affiliations:** ^1^ Institut Gustave Roussy Villejuif France; ^2^ H. Lee Moffitt Cancer Center & Research Institute Tampa Florida USA; ^3^ Candiolo Cancer Institute FPO‐IRCCS Turin Italy; ^4^ Feinberg School of Medicine Northwestern University Chicago Illinois USA; ^5^ West Cancer Center Memphis Tennessee USA; ^6^ Humanitas Clinical and Research Center IRCCS Humanitas University Rozzano‐Milano Italy; ^7^ IRCCS Istituto Nazionale dei Tumori University of Milano Milano Italy; ^8^ Sarah Cannon Research Institute Nashville Tennessee USA; ^9^ Stanford Cancer Institute Stanford California USA; ^10^ Centre Leon Berard Lyon France; ^11^ Cross Cancer Institute Edmonton Canada; ^12^ Division of Hematology/Oncology University of Wisconsin Madison Wisconsin USA; ^13^ Yale Cancer Center New Haven Connecticut USA; ^14^ Bristol Myers Squibb Princeton New Jersey USA; ^15^ Division of Medical Oncology and Hematology Princess Margaret Cancer Centre University of Toronto Toronto Canada

**Keywords:** new drug development, non‐Hodgkin lymphoma, phase 1 clinical trials

## Abstract

There is a need for additional treatment options for patients with relapsed or refractory diffuse large B‐cell lymphoma (DLBCL) who do not benefit from available therapies. We examined combinations of the cereblon E3 ligase modulator (CELMoD) agent avadomide (CC‐122), the selective, ATP‐competitive mammalian target of rapamycin kinase inhibitor CC‐223, and the potent, selective, covalent Bruton tyrosine kinase inhibitor CC‐292 in patients with relapsed/refractory (R/R) DLBCL.

In the multicenter, phase Ib CC‐122‐DLBCL‐001 study (NCT02031419), the dose‐escalation portion explored combinations of CC‐122, CC‐223, and CC‐292 administered as doublets or triplets with rituximab in patients with chemorefractory DLBCL. Primary endpoints were safety, tolerability, and dose‐limiting toxicities; additional endpoints included pharmacokinetics, pharmacodynamics, biomarkers, and preliminary efficacy.

As of December 1, 2017, 106 patients were enrolled across four cohorts. The median age was 65 years (range 24–84 years), and patients had a median of 3 (range 1–10) prior to regimens. A total of 101 patients (95.3%) discontinued, most commonly due to disease progression (49.1%). The most common any‐grade adverse events (AEs) across treatment arms were gastrointestinal and hematologic; the most common grade 3/4 AEs were hematologic. CC‐122 was well tolerated, with no unexpected safety concerns. Preliminary efficacy was observed in three of four treatment arms. CC‐122 plus rituximab was considered suitable for dose expansion, whereas CC‐223 and CC‐292 combinations were associated with enhanced toxicity and/or insufficient improvement in responses.

CC‐122 plus rituximab was well tolerated, with preliminary antitumor activity in patients with R/R DLBCL. This innovative study demonstrates the feasibility of assessing the tolerability and preliminary efficacy of novel combinations utilizing a multi‐arm dose‐finding design.

## 1 INTRODUCTION

Non‐Hodgkin lymphoma (NHL) comprises a heterogeneous group of lymphoproliferative diseases with differing patterns of clinical presentation and responses to treatment [1, 2]. Diffuse large B‐cell lymphoma (DLBCL) is characterized by aggressive clinical behavior and is the most common type of NHL, accounting for approximately 40% of diagnoses [2, 3]. The standard first‐line treatment for DLBCL is the regimen R‐CHOP (cyclophosphamide, doxorubicin, vincristine, and prednisone plus the CD20‐directed monoclonal antibody [mAb] rituximab) [4]. Current guidelines recommend cytotoxic chemotherapy plus rituximab followed by autologous stem cell transplant (ASCT) in eligible patients as salvage treatment after first relapse [4]. However, those patients with DLBCL whose disease is refractory to salvage therapy and/or are ineligible for transplantation, or whose disease relapses after ASCT, have historically had few treatment options and typically have a poor prognosis [5, 6, 7, 8]. A number of therapies have recently demonstrated efficacy in the third‐line treatment setting and are now standard of care, including anti‐CD19 chimeric antigen receptor (CAR) T‐cell therapies, the anti‐CD19 antibody‐drug conjugate (ADC) loncastuximab tesirine, and the nuclear export inhibitor selinexor [4]. Initial data from two studies of anti‐CD19 CAR T‐cell therapies as second‐line therapy in ASCT‐eligible patients (TRANSFORM NHL‐001 and ZUMA‐9) have also shown improved event‐free survival and response rates compared with high‐dose chemotherapy and ASCT, suggesting that new therapies may offer improvements in outcome in earlier treatment lines as well [9, 10].

Various targeted therapies have demonstrated single‐agent activity in relapsed/refractory (R/R) DLBCL, including the immunomodulatory drug (iMiD) agent lenalidomide [11, 12], the Bruton tyrosine kinase (BTK) inhibitor ibrutinib [13], and the mammalian target of rapamycin (mTOR) inhibitor temsirolimus [14]. These therapies target signaling pathways involved in B‐cell growth and survival, and there is considerable interest in investigating combinations containing these drugs or compounds with similar mechanisms of action for the treatment of NHL [15]. To date, promising results have been observed with some combinations, including lenalidomide plus rituximab in follicular lymphoma (FL), lenalidomide plus tafasitamab in R/R DLBCL, everolimus plus R‐CHOP in first‐line DLBCL, and the triplet of ibrutinib, lenalidomide, and rituximab in first‐line DLBCL [16, 17, 18, 19].

A greater understanding of the pathology of DLBCL has led to the identification of molecular subgroups that are associated with distinct actionable targets and differential responses to some therapies, notably double‐hit lymphoma and the germinal center B‐cell (GCB) and activated B‐cell (ABC) subtypes [20, 21]. For example, the ECOG‐ACRIN‐1412 trial, which compared lenalidomide in combination with R‐CHOP to R‐CHOP alone, found that the addition of lenalidomide was associated with improved progression‐free survival (PFS) in patients with ABC subtype DLBCL, but not in patients with the GCB subtype [22]. Knowledge of actionable targets associated with specific subtypes allows the selection of rational drug combinations with complementary mechanisms of action that are likely to have enhanced antitumor activity [23]. However, clinical investigation of such combinations must be done with care, due to the possibility that additive or synergistic effects may result in increased toxicity [20, 23]. An example of such toxicities was reported from a study evaluating the combination of idelalisib, a phosphatidylinositol‐3‐kinase (PI3K) *δ* inhibitor, lenalidomide, and rituximab in mantle cell and FL [24]. Although doublets of idelalisib or lenalidomide with rituximab have previously demonstrated efficacy and safety in clinical trials, the combination of idelalisib and lenalidomide, with and without rituximab, was unexpectedly found to be poorly tolerated [24].

Avadomide (CC‐122) is a novel cereblon E3 ligase modulator (CELMoD) agent that binds to cereblon in the cullin4 E3 ubiquitin ligase complex, leading to ubiquitination and subsequent degradation of the hematopoietic transcription factors Aiolos and Ikaros [25]. The degradation of Aiolos and Ikaros results in derepression of interferon‐response gene promoters and apoptosis in malignant B cells and derepression of interleukin‐2 expression and secretion in T cells, resulting in T‐cell activation [25, 26, 27, 28]. CC‐122 was well tolerated and demonstrated promising preliminary activity as monotherapy in R/R DLBCL [29, 30, 31], as well as preliminary activity in combination with the anti‐CD20 mAb obinutuzumab in DLBCL [31]. A novel gene expression classifier has also been developed that identified a subgroup of patients with an improved clinical response to CC‐122 monotherapy [29, 32]. CC‐223 is a selective, ATP‐competitive inhibitor of mTOR, a kinase that plays a key role in the regulation of cell proliferation and survival [33, 34, 35]. CC‐292 is a potent, selective, covalent inhibitor of BTK, a critical component of the B‐cell receptor signaling network required for B‐cell development [36].

As iMiD agents, mTOR/PI3K pathway inhibitors, and BTK inhibitors have demonstrated benefit as monotherapies and in combination with other agents, it is reasonable to suggest that combinations of CC‐122, CC‐223, and CC‐292 may provide additional benefit over monotherapies. Rituximab has also been shown to enhance the antitumor activity of agents with these mechanisms of action in clinical studies [37, 38, 39], providing a rationale for the evaluation of rituximab in combination with all three agents.

The rationale for the addition of rituximab is further supported by preclinical studies, including enhancement of rituximab‐mediated antibody‐dependent cell‐mediated cytotoxicity by CC‐122 in two DLBCL cell lines (Figure S1) and increased tumor growth inhibition (TGI) with CC‐122 plus rituximab compared with either agent alone in a DLBCL xenograft model (55% TGI with combination therapy versus 28% and 19% TGI for CC‐122 and rituximab monotherapies, respectively; Figure S1).

The CC‐122‐DLBCL‐001 study was conducted to evaluate novel combinations of these agents administered as doublets and triplets in combination with rituximab in patients with R/R DLBCL in dose escalation and DLBCL or FL in dose expansion. Here, we report results from patients with DLBCL treated in the dose‐escalation (part A) portion of the study.

## 2 METHODS

### 2.1 Study design and patients

**FIGURE 1:**
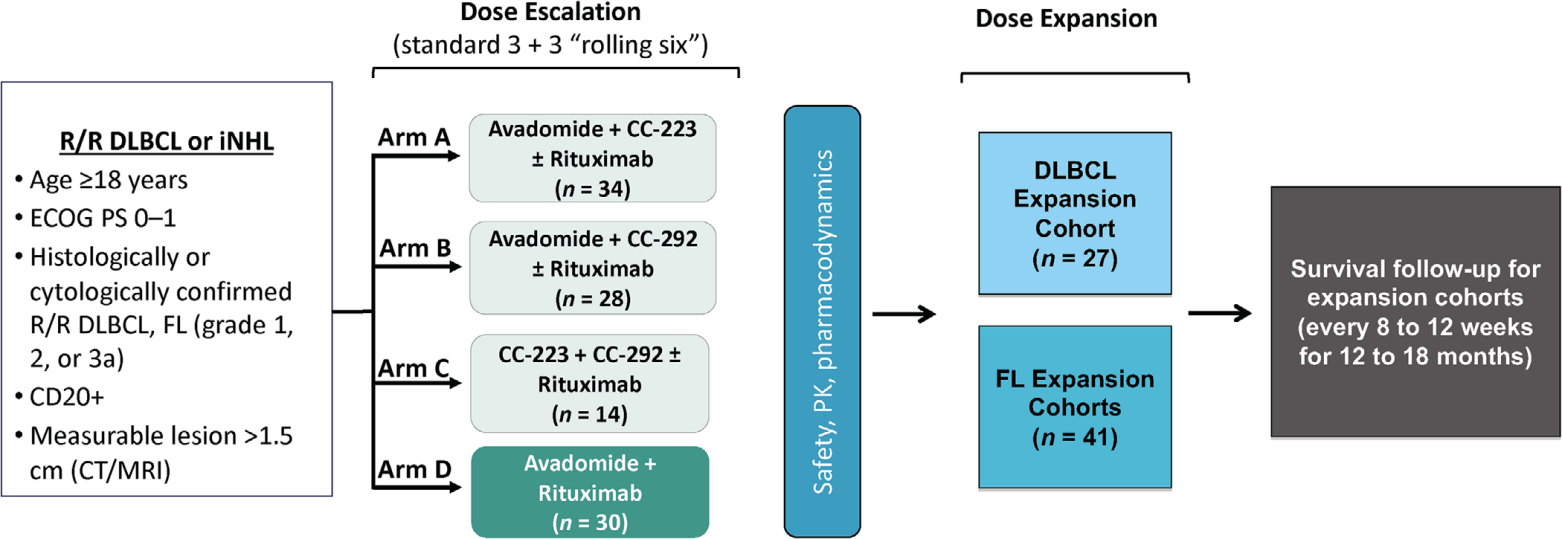
CC‐122‐DLBCL‐001 study design: CC‐122‐DLBCL‐001 is a multicenter, open‐label, phase Ib study of avadomide, CC‐223, and CC‐292 administered orally as doublets or triplets in combination with rituximab, as well as an avadomide plus rituximab doublet in patients with R/R DLBCL or FL. The dose‐escalation part of the study included patients with DLBCL only. CT/MRI, computed tomography/magnetic resonance imaging; DLBCL, diffuse large B‐cell lymphoma; ECOG PS, Eastern Cooperative Oncology Group performance status; FL, follicular lymphoma; iNHL, indolent non‐Hodgkin lymphoma; PK, pharmacokinetics; R/R, relapsed/refractory

CC‐122‐DLBCL‐001 (NCT02031419, 2013‐001501‐81) is a multicenter, open‐label, phase Ib study with a dose‐escalation phase reported here and a dose‐expansion phase. Exploration of doses for each novel agent used a standard 3 + 3 dose design [40], with a fixed dose of rituximab in higher‐dose cohorts (Figure 1). The study was conducted in accordance with the Declaration of Helsinki and in adherence to Good Clinical Practice as described in the International Council for Harmonisation E6 guidelines. The protocol was reviewed and approved by each site's Institutional Review Board or Independent Ethics Committee before initiation of the study, and all patients provided written informed consent.

The primary objectives were to determine the safety and tolerability of oral CC‐122, CC‐223, and CC‐292 when administered as doublets and triplets in combination with rituximab, to assess the safety and tolerability of CC‐122 plus rituximab, and to define the non‐tolerated dose (NTD), the maximum‐tolerated dose (MTD), and/or the recommended phase 2 dose (RP2D) of each combination. Secondary objectives included characterization of steady‐state pharmacokinetics (PK) of CC‐122, CC‐223, and CC‐292 combinations following oral administration and assessment of preliminary efficacy. Exploratory objectives included evaluating blood pharmacodynamic biomarkers of CC‐122 and the relationship between these markers and clinical activity, characterizing the steady‐state PK of the M1 metabolite of CC‐223 administered in combination with CC‐292, and evaluating baseline tumor biomarkers and the effects of study treatments on them.

Eligible patients were aged > 18 years, with histologically or cytologically confirmed R/R DLBCL (including transformed indolent lymphoma). All patients had chemorefractory DLBCL, defined as having a stable disease or progressive disease (PD) as the best response to their last chemotherapy and/or PD or recurrence within 12 months of prior ASCT. Patients must have received an anti‐CD20 mAb therapy (unless the tumor was CD20‐negative) and anthracycline‐containing chemotherapy as well as ≥1 prior salvage treatment (unless ineligible for ASCT). See Supporting Information for additional eligibility criteria.

### 2.2 Treatment

CC‐122 hydrochloride, referred to here as CC‐122, as an active pharmaceutical ingredient in capsule (AIC) formulation, was administered orally once daily (QD). An alternate intermittent schedule of CC‐122 QD for five of every seven consecutive days (5/7), which improved tolerability and reduced the frequency and severity of neutropenia in a study of CC‐122 monotherapy in DLBCL, was also explored [29]. CC‐292 was administered orally twice daily (BID) and CC‐223 was administered orally QD. Rituximab was administered on day 8 of cycle 1, day 1 of cycles 2–6, and day 1 of every third cycle thereafter. Treatment arms are shown in Figure S2. Patients in arm A received CC‐122 and CC‐223 with or without rituximab (starting at CC‐122 2 mg QD and CC‐223 20 mg QD), patients in arm B received CC‐122 plus CC‐292 with or without rituximab (starting at CC‐122 2 mg QD and CC‐292 500 mg BID), patients in arm C were treated with CC‐223 and CC‐292 (starting at CC‐223 20 mg QD and CC‐292 500 mg BID), and those in arm D received CC‐122 plus rituximab (starting at CC‐122 2 mg QD). In arm D, a CC‐122 3 mg formulated capsule [29] was introduced with the same strength as CC‐122 3 mg AIC. Each dose level was cleared before the next was started. If unacceptable toxicity occurred at the initial dose level, dose reductions for CC‐122 (1.5 mg QD and 1 mg QD) and CC‐223 (15 mg QD) were allowed; no starting dose reductions for CC‐292 were planned. For arm A, the Safety Review Committee (SRC) determined which of the two drugs in the doublet to reduce. For arms B and D, the CC‐122 dose was reduced and for arm C, the CC‐223 dose was reduced. Assignment of patients to study arms was at the discretion of the investigator and based on slot availability per the study design.

Patients who experienced dose‐limiting toxicity (DLT) after receiving ≥1 dose of study treatment or those who received ≥80% of planned doses of CC‐122, CC‐223, CC‐292, and rituximab during cycle 1 without experiencing a DLT were considered DLT‐evaluable. If zero out of ≥3 or ≤1 out of six evaluable patients in a cohort experienced a DLT during cycle 1 in parallel dose cohorts, then dose escalation could occur. DLTs are defined in the Supplementary Materials; individual investigators performed response assessments and determined DLTs. The NTD was defined as the dose level at which ≥2 of six evaluable patients in a cohort experienced a DLT during cycle 1. The MTD was defined as the last dose level below the NTD at which ≤1 of 6 evaluable patients had a DLT during cycle 1. During cycle 1, any adverse event (AE) that led to dose reduction was considered a DLT. Patients could resume CC‐122 at a reduced dose only if they recovered to grade ≤1 within 14 days of dose interruption; two dose reductions were allowed before the patient was withdrawn from the study. Dose reductions were permitted for CC‐122, CC‐223, and CC‐292; no dose reductions were allowed for rituximab, but treatment could be discontinued at the discretion of the investigator. After cycle 1, the dose level could be increased if the alternative dose level was well tolerated in ≥1 cohort of other patients. For arms A, B, and D, CC‐122 dosing was modified to the intermittent 5/7‐day schedule based on SRC review and escalated until the MTD/RP2D was established. Treatment was given until disease progression, unacceptable toxicity, or patient/physician decision to withdraw consent.

### 2.3 Study assessments

AEs were assessed according to the National Cancer Institute Common Terminology Criteria for AEs, version 4.03, up to 28 days after the last treatment. Blood samples for PK analysis were collected on days 15 to 28, predose, and at 1.5 and 3 h post‐dose on days 1 and 15 of cycle 1 for all dose cohorts. Additional blood samples were collected on day 15 from patients in dose level 2 of arm C to examine CC‐223 and CC‐292 steady‐state PK. Pharmacodynamic biomarkers of each novel agent were explored at baseline and cycle 1, day 1 (pre‐dose, and at 1.5 and 3 h) and cycle 1, day 15 (pre‐dose, and at 1.5 and 3 h), including cereblon modulation substrates in B and T cells in patients treated with CC‐122 and mTOR signaling pathway biomarkers (e.g., p4EBP1 and pAKT) in patients treated with CC‐223. Tumor assessments were performed at screening and at the end of even‐numbered cycles through cycle 6, then every three cycles through cycle 12, and every 6 months thereafter. Responses were assessed by the investigator per International Working Group 2007 criteria [41].

### 2.4 Statistical analyses

Safety analyses were performed on all patients who received ≥1 dose of study treatment (safety population). The PK population comprised all patients who took ≥1 dose of study treatment and had ≥1 measured drug concentration. Efficacy‐evaluable patients were those who completed ≥1 treatment cycle and had a baseline and ≥1 postbaseline efficacy assessment. The biomarker‐evaluable population comprised all patients who took ≥1 dose of study treatment and had ≥1 pharmacodynamic assessment. Safety and efficacy analyses were based on the safety population except where noted (PK and biomarkers). PK parameters were calculated using actual times relative to the most recent administration. The median duration of response (DOR) and median PFS were calculated per the Kaplan‐Meier method.

## 3 RESULTS

### 3.1 Patients and treatment

From December 18, 2013, to June 21, 2016, 106 patients with DLBCL were enrolled across the dose‐escalation cohorts, including 34 in arm A, 28 in arm B, 14 in arm C, and 30 in arm D. As of the cutoff date (December 1, 2017), 101 patients (95.3%) had discontinued study treatment and five (4.7%) were ongoing (Figure S2). The most common reasons for discontinuation were PD (*n* = 52 [49.1%]), AEs (*n* = 19 [17.9%]), death due to disease under study or complications thereof (*n* = 15 [14.2%]), and death due to AE (*n* = 1 [< 1.0%]; cardiac arrest unrelated to study treatment). Patient demographics and baseline characteristics are shown in Table 1. The overall median age was 65 years (range 24–84 years), and 59.4% were male. At enrollment, most patients (56.6%) had disease stage IV, 31.4% had transformed DLBCL, 14.7% had a double‐hit disease, and eight patients (7.5%) had progression of disease within 12 months of diagnosis (POD12). The cell of origin (COO) was not determined for patients enrolled in the dose‐escalation portion of the study. Patients received a median of 3 (range 1–10) prior systemic anticancer therapies; 72 (70.6%) received >2 prior therapies, and 31 (30.4%) had received prior ASCT. A majority of patients received prior therapy with rituximab (97.1%). No patients had previously received CAR T‐cell therapies.

**TABLE 1:** Patient baseline characteristics

	**Arm A**	**Arm B**	**Arm C**	**Arm D**	
**Characteristic**	**Avadomide + CC‐223 ± R (*n* = 34)**	**Avadomide + CC‐292 ± R (*n* = 28)**	**CC‐292 + CC‐223 ± R (*n* = 14)**	**Avadomide + R (*n* = 30)**	**Overall (*N* = 106)**
Median age, years (range)	63 (28–84)	57 (24–84)	68 (25–80)	66 (28–84)	65 (24–84)
Age > 65 years, *n* (%)^a^	13 (38.2)	8 (28.6)	9 (64.3)	15 (50.0)	45 (42.5)
Male, *n* (%)^a^	19 (55.9)	17 (60.7)	8 (57.1)	19 (63.3)	63 (59.4)
ECOG PS, *n* (%)^a^					
0	14 (41.2)	18 (64.3)	6 (42.9)	11 (36.7)	49 (46.2)
1	17 (50.0)	9 (32.1)	8 (57.1)	19 (63.3)	53 (50.0)
Missing	3 (8.8)	1 (3.6)	0	0	4 (3.8)
Disease stage, *n* (%)					
I	1 (3.2)	0	0	0	1 (1.0)
II	6 (17.6)	3 (10.7)	1 (7.1)	4 (13.3)	14 (13.2)
III	8 (23.5)	6 (21.4)	4 (28.6)	6 (20.0)	24 (22.6)
IV	15 (44.1)	16 (57.1)	9 (64.3)	20 (66.7)	60 (56.6)
Missing	1 (3.2)	2 (7.4)	0	0	3 (2.9)
Transformed DLBCL, *n* (%)	11 (35.5)	9 (33.3)	4 (28.6)	8 (26.7)	32 (31.4)
Double‐hit, *n* (%)^b^	5 (16.1)	5 (18.5)	1 (7.1)	4 (13.3)	15 (14.7)
Bone marrow involvement, *n* (%)	5 (16.1)	3 (11.1)	2 (14.2)	4 (13.3)	14 (13.7)
Median no. prior systemic anticancer regimens (range)	3 (2‐8)	3 (1–7)	3 (1–5)	3 (2–10)	3 (1–10)
1	0	3 (11.1)	2 (14.3)	0	5 (4.9)
2	7 (22.6)	6 (22.2)	4 (28.6)	8 (26.7)	25 (24.5)
>2	24 (77.4)	18 (66.7)	8 (57.1)	22 (73.3)	72 (70.6)
Prior rituximab, *n* (%)	29 (93.5)	27 (100)	13 (92.9)	30 (100)	99 (97.1)
Prior ASCT, *n* (%)	11 (35.5)	9 (33.3)	3 (21.4)	8 (26.7)	31 (30.4)
POD12, *n* (%)	5 (14.7)	2 (7.1)	0	1 (3.3)	8 (7.5)
Median time since last recurrence (range), months	1.0 (‐1.0–5.7)	0.5 (0–4.9)	1.1 (0.3–10.6)	0.9 (‐0.1–3.8)	0.9 (‐1.0–10.6)

Abbreviations: ASCT, autologous stem cell transplant; DLBCL, diffuse large B‐cell lymphoma; ECOG PS, Eastern Cooperative Oncology Group performance status; POD12, progression of disease within 12 months of diagnosis; R, rituximab.

^a^ Data in the enrolled population shown; all other results are in the safety population.

^b^ Rearrangement or overexpression of both *BCL2* and *MYC*.

The sequence of the dose levels explored in each arm is shown in Table S1. Among the 88 patients across treatment arms who received CC‐122, the median duration of CC‐122 treatment was 56 days (range 7–941 days) and the median relative CC‐122 dose intensity was 1.0 (range 0.5–1.0; Table S2). Eighteen patients (20.5%) received CC‐122 dose reductions (10 patients in arm A, three patients in arm B, and five patients in arm D); 11 due to AEs. Among the 45 patients treated with CC‐223, the overall median CC‐223 treatment duration was 56 days (range 7–937 days) and the median relative dose intensity was 1.0 (range 0.6–1.0). Eleven patients (24.4%) had dose reductions of CC‐223; eight due to AEs. Among the 41 patients who received CC‐292, the median duration of CC‐292 treatment was 56 days (range 7–542 days) and the median relative dose intensity was 1.0 (range 0.6–1.0). Of the 16 patients (39.0%) who had CC‐292 dose reductions; eight were due to AEs. Sixty‐one patients received rituximab for an overall median duration of 56 days (range 28–980 days), and the median relative dose intensity was 1.0 (range 0.5–1.1). Details of dose reductions and interruptions for each drug and treatment arm are shown in Table S2.

### 3.2 Safety

Treatment‐emergent AEs (TEAEs) occurring in ≥10% of patients by treatment arm are reported in Table 2. The most common TEAEs of any grade across treatment arms were gastrointestinal (GI) and hematologic events. Diarrhea was the most common any‐grade TEAE, particularly in arms A (67.7%), B (44.4%), and C (64.3%); neutropenia (50.0%) was the most common any‐grade hematologic TEAE, particularly in arms A (48.4%), B (44.4%), and D (50.0%). In all treatment arms, the most common grade 3/4 TEAEs were hematologic.

**TABLE 2:** Treatment‐emergent adverse events by treatment arm (safety population)^a^

	**Arm A**		**Arm B**		**Arm C**		**Arm D**	
	**Avadomide + CC‐223 ± R (*n* = 31)**		**Avadomide + CC‐292 ± R (*n* = 27)**		**CC‐292 + CC‐223 (*n* = 14)**		**Avadomide + R (*n* = 30)**	
	**Any grade**	**Grade 3/4**	**Any grade**	**Grade 3/4**	**Any grade**	**Grade 3/4**	**Any grade**	**Grade 3/4**
Any TEAE, *n* (%)	31 (100)	27 (87.1)	27 (100)	25 (92.6)	14 (100)	14 (100)	30 (100)	22 (73.3)
**Hematologic**								
Neutropenia	15 (48.4)	14 (45.2)	12 (44.4)	12 (44.4)	3 (21.4)	2 (14.3)	15 (50.0)	11 (36.7)
Anemia	9 (29.0)	4 (12.9)	6 (22.2)	5 (18.5)	2 (14.3)	2 (14.3)	8 (26.7)	–
Thrombocytopenia	7 (22.6)	5 (16.1)	11 (40.7)	7 (25.9)	5 (35.7)	4 (28.6)	5 (16.7)	4 (13.3)
Febrile neutropenia	3 (9.7)	3 (9.7)	5 (18.5)	5 (18.5)	–	–	2 (6.7)	2 (6.7)
**Gastrointestinal**								
Diarrhea	21 (67.7)	6 (19.4)	12 (44.4)	–	9 (64.3)	2 (14.3)	3 (10.0)	–
Abdominal pain	7 (22.6)	–	8 (29.6)	–	–	–	–	–
Nausea	6 (19.4)	–	8 (29.6)	–	3 (21.4)	–	5 (16.7)	–
Vomiting	6 (19.4)	–	4 (14.8)	–	3 (21.4)	–	3 (10.0)	–
Constipation	4 (12.9)	–	3 (11.1)	–	–	–	6 (20.0)	–
Dyspepsia	–	–	4 (14.8)	–	3 (21.4)	–	–	–
**Other**								
Hypokalemia	12 (38.7)	3 (9.7)	3 (11.1)	–	3 (21.4)	–	–	–
Pyrexia	12 (38.7)	–	10 (37.0)	–	4 (28.6)	–	10 (33.3)	–
Fatigue	12 (38.7)	2 (6.5)	8 (29.6)	2 (7.4)	2 (14.3)	–	3 (10.0)	–
Cough	11 (35.5)	–	4 (14.8)		3 (21.4)	–	8 (26.7)	–
Dyspnea	11 (35.5)	–	7 (25.9)	2 (7.4)	–	–	8 (26.7)	–
Rash	11 (35.5)	–	3 (11.1)	–	2 (14.3)	–	5 (16.7)	–
Decreased appetite	9 (29.0)	–	–	–	–	–	7 (23.3)	–
Hyperglycemia	9 (29.0)	2 (6.5)	–	–	2 (14.3)	–	–	–
Overdose	7 (22.6)	–	3 (11.1)	–	2 (14.3)	–	–	–
Blood creatinine increased	6 (19.4)	–	3 (11.1)	–	2 (14.3)	–	–	–
Proteinuria	6 (19.4)	–	–	–	–	–	–	–
Pruritus	5 (16.1)	–	3 (11.1)	–	–	–	8 (26.7)	–
Asthenia	5 (16.1)	2 (6.5)	–	–	4 (28.6)	3 (21.4)	5 (16.7)	2 (6.7)
Urinary tract infection	5 (16.1)	–	4 (14.8)	–	–	–	4 (13.3)	–
Dizziness	5 (16.1)	–	–	–	–	–	3 (10.0)	–
Edema peripheral	4 (12.9)	–	6 (22.2)	–	4 (28.6)	–	4 (13.3)	–
AST increased	4 (12.9)	–	5 (18.5)	–	–	–	3 (10.0)	–
Hypotension	4 (12.9)	–	–	–	5 (35.7)	4 (28.6)	3 (10.0)	2 (6.7)
General health deterioration^b^	–	–	8 (29.6)	5 (18.5)	2 (14.3)	2 (14.3)	4 (13.3)	–
Noncardiac chest pain	–	–	4 (14.8)	3 (11.1)	–	–	–	–
Hyperbilirubinemia	–	–	4 (14.8)	–	–	–	–	–
Pneumonia	–	–	4 (14.8)	–	–	–	–	2 (6.7)
Headache	–	–	4 (14.8)	–	–	–	–	–

Abbreviations: AST, aspartate aminotransferase; R, rituximab; TEAE, treatment‐emergent adverse event.

^a^ Events of any grade occurring in ≥15% of patients and grade 3/4 events occurring in ≥5% of patients by treatment arm are reported. Dashes indicate an incidence not meeting these cutoffs.

^b^ General health deterioration included clinical disease progression or worsening health status; patients did not meet the criteria for radiographic disease progression.

In arm A, GI disorders showed a trend suggesting a dose effect, while other TEAEs (including neutropenia and thrombocytopenia) showed improvement with intermittent dosing of CC‐122 (Table S3). A potential dose effect was also observed in arm B; however, modest improvement in neutropenia, thrombocytopenia, pyrexia, and diarrhea was observed with intermittent dosing of CC‐122. Among the most common TEAEs in arm C, diarrhea and thrombocytopenia showed a trend suggesting a dose‐effect. The most common TEAEs in arm D (hematologic, general disorders, and GI disorders) showed improvement with intermittent dosing of CC‐122. Most patients had ≥1 TEAE suspected of being drug‐related (Table S4).

At least 50% of patients in each arm reported a serious AE (SAE); however, the occurrence of SAEs in >1 patient was uncommon (Table S5). Infections were among the most common SAEs in each arm (arm A, 19.4%; arm B, 11.1%; arm C, 7.1%; and arm D, 20.0%). SAEs related to the study drug were observed in eight patients (25.8%) in arm A, nine patients (33.3%) in arm B, four patients (28.6%) in arm C, and three patients (10.0%) in arm D. Overall, 14 patients (13.7%) died, none while on treatment but all within 28 days of the last dose of study drug. Of these, 13 patients died from disease progression; the remaining patient (in arm D) died from a TEAE (cardiac arrest unrelated to study treatment).

Among the DLT‐evaluable patients, four (15.4%) in arm A, eight (33.3%) in arm B, four (40.0%) in arm C, and two (8.3%) in arm D experienced a DLT. A summary of DLTs by dose level and treatment arm is presented in Table S6. A lower proportion of patients in arms A and D experienced DLTs relative to arms B and C. The MTD for arm A was not established as the MTD/RP2D for CC‐122 monotherapy was reached in a previous clinical study [30]. However, a decision was made not to expand this arm, and the cohort was closed. The MTD for arm B was determined to be CC‐122 1 mg QD for 5/7 days plus CC‐292 500 mg BID and 375 mg/m^2^ rituximab. Arm B was not expanded, because further development of CC‐292 was terminated. The MTD for arm C was not established; both dose levels evaluated were determined to be NTDs, and the cohort was closed to enrollment. In arm D, CC‐122 3 mg in a formulated capsule, given QD for 5/7 days plus 375 mg/m^2^ rituximab was considered suitable for expansion.

### 3.3 Efficacy

**FIGURE 2:**
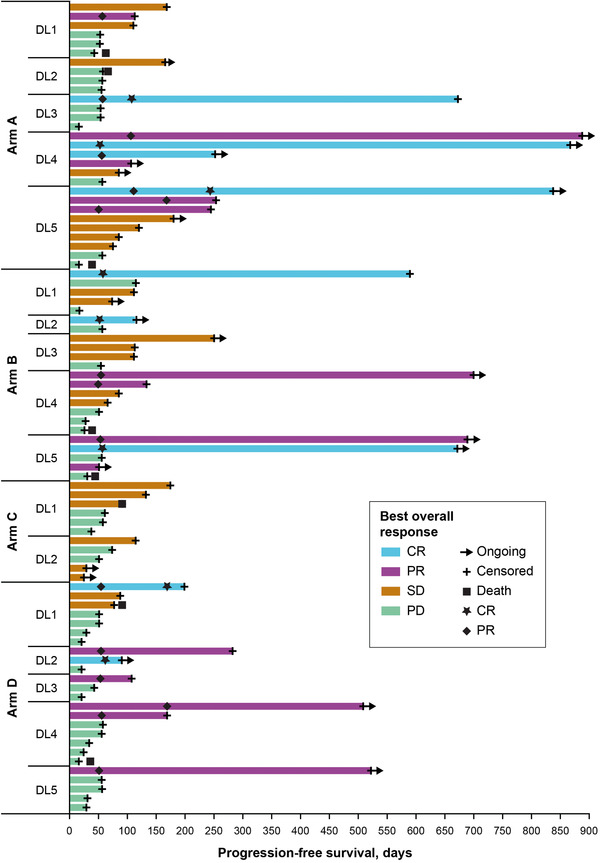
Progression‐free survival and best overall response (safety population). Swimplot presents duration of progression‐free survival and best overall response by arm and dose level. CR, complete response; DL, dose level; NE, not estimable; PD, progressive disease; PR, partial response; SD, stable disease

**T**he objective response rate (ORR) among treated patients was 29.0% (95% confidence interval [CI], 14.2–48.0) in arm A, 25.9% (11.1–46.3) in arm B, 0% in arm C, and 23.3% (9.9–42.3) in arm D (Table 3). Complete responses (CRs) occurred in four patients (12.9%) in arm A, three (11.1%) in arm B, and two (6.7%) in arm D. The addition of rituximab appeared to improve response rates in arms A and B (Table S7). There appeared to be a dose‐dependent effect on clinical response in arms A and B. In arm A, the ORR was 12.5% (2/16 patients) in patients treated with 2 mg CC‐122 (dose levels 1, 2, and 3) compared with 46.7% (7/15 patients) in patients treated with higher doses of CC‐122 (3 and 4 mg [dose levels 4 and 5]). In arm B, the ORR was 15.8% (3/19 patients) in patients treated with 1 mg of CC‐122 (dose levels 1, 3, and 4) and 50% (4/8 patients) in those treated with 2 mg of CC‐122 (dose levels 2 and 5) (Table S7). Despite similar ORRs in arms A, B, and D, the median response duration was >3 times longer in arms A and B (616 days and not reached, respectively) than in arm D (183 days). Across treatment arms, of the responders, three proceeded to ASCT. The median PFS and 6‐ and 12‐month estimated PFS rates were substantially higher in arms A and B relative to arms C and D (Table 3). Prolonged responses of >500 days were observed in 10 patients (Figure 2).

**TABLE 3:** Efficacy by treatment arm (safety population)

	**Arm A**	**Arm B**	**Arm C**	**Arm D**
	**Avadomide + CC‐223 ± R (*n* = 31)**	**Avadomide + CC‐292 ± R (*n* = 27)**	**CC‐292 + CC‐223 (*n* = 14)**	**Avadomide + R (*n* = 30)**
ORR, *n* (%) [95% CI]^a^	9 (29.0) [14.2–48.0]	7 (25.9) [11.1–46.3]	0 [NA]	7 (23.3) [9.9–42.3]
CR	4 (12.9)	3 (11.1)	0	2 (6.7)
PR	5 (16.1)	4 (14.8)	0	5 (16.7)
SD	8 (25.8)	7 (25.9)	6 (42.9)	2 (6.7)
PD	12 (38.7)	9 (33.3)	5 (35.7)	16 (53.3)
Missing	2 (6.5)	4 (14.8)	3 (21.4)	5 (16.7)
ORR by subgroup, *n*/*N* (%)				
Double‐hit	1/5 (20.0)	0/5	0/1	1/4 (25.0)
No double‐hit	8/26 (30.8)	7/22 (31.8)	0/13	6/26 (23.1)
Prior ASCT	4/11 (36.4)	3/9 (33.3)	0/3	2/8 (25.0)
No prior ASCT	5/20 (25.0)	4/18 (22.2)	0/11	5/22 (22.7)
Median PFS, *d* (95% CI)	110.0 (57.0–246.0)	111.0 (54.0–132.0)	66.5 (31.0–113.0)	50.0 (29.0–77.0)
6‐mo PFS, % (95% CI)	35.4 (18.1–53.1)	26.8 (10.7–46.0)	0	17.0 (5.7–33.5)
12‐mo PFS, % (95% CI)	25.3 (10.2–43.6)	26.8 (10.7–46.0)	0	8.5 (1.5–23.4)
Median DOR, *d* (95% CI)	616 (57–NA)	NR (83–NR)	NA	183 (55–NR)

Abbreviations: CR, complete response; DOR, duration of response; NA, not applicable; NR, not reached; ORR, objective response rate; PD, progressive disease; PFS, progression‐free survival; PR, partial response; R, rituximab; SD, stable disease.

^a^ Response as determined by the investigator based on International Working Group Criteria for malignant lymphoma.^33.^

### 3.4 Biomarker analysis

In a prior clinical study, Aiolos was shown to be a dose‐dependent blood pharmacodynamic biomarker of CC‐122 [29, 30]. Following administration of 1–4 mg of CC‐122 in combination with CC‐223 or CC‐292, with or without rituximab, Aiolos degradation was observed, indicating target engagement and pharmacologic activity. In arms B and D, there was a dose‐dependent trend in decreased Aiolos levels in CD3+ T cells and CD19+ B cells from baseline to cycle 1 day 1 at 3 h post‐dose (Figure 3). Aiolos degradation in CD3+ T cells from equivalent 4‐mg CC‐122 doses in arms A and D identified a potential antagonistic influence of CC‐223 on CC‐122‐mediated degradation of Aiolos, which resulted in a 14% decrease in Aiolos in arm A compared with a 46% decrease in arm D (rituximab‐containing regimen). Data from arm B support a trend towards increased Aiolos degradation in patients receiving either 1 or 2 mg of CC‐122 at 3 h post‐dose. Among patients treated with 1 mg of CC‐122, the median decline in Aiolos levels was approximately 20% in CD3+ T cells and 5% in CD19+ B cells, respectively. In the 2‐mg CC‐122 group, there was a decline of approximately 40% in CD3+ T cells and 32% in CD19+ B cells. Furthermore, the combined data indicate that pharmacodynamic effects of CC‐122 observed in arm B were similar to those obtained with higher doses of CC‐122 in arms A and D, suggesting that the BTK inhibitor CC‐292 may potentiate the activity of CC‐122 to increase the extent of Aiolos degradation.

FIGURE 3Per cent change from baseline in Aiolos in CD3+ T cells (A) and CD19+ B cells (B) and in p4EBP1 (C) and pAKT (D) at 3 h post‐dose on day 1 of cycle 1 (biomarker‐evaluable population). The change in Aiolos expression in CD3+ T cells and CD19+ B cells was calculated within each avadomide‐ and CC‐223‐containing arm. *p ≤ 0.05 versus baseline; **p ≤ 0.01 versus baseline; ***p ≤ 0.001 versus baseline; DL, dose level; MEFL, molecules of equivalent fluorescence label; NS, not significant

**Figure:**
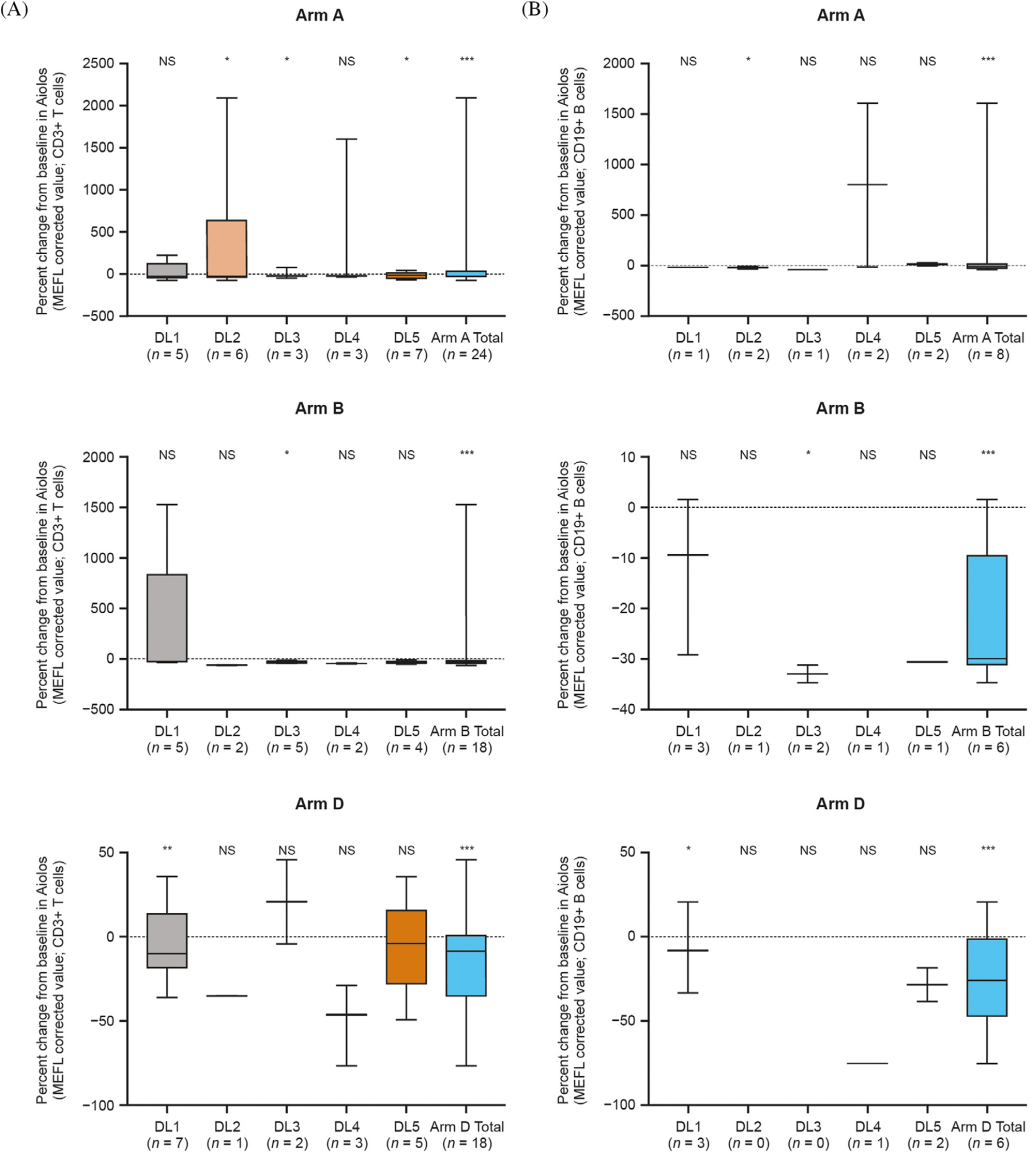

**Figure:**
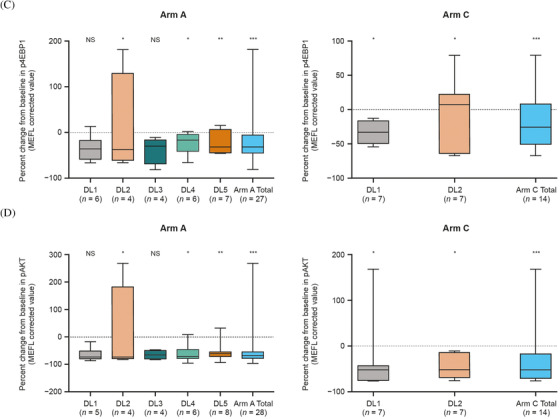

Following administration of CC‐223, a dramatic decrease in levels of the mTOR signaling pathway biomarkers p4EBP1 and pAKT within 3 h on cycle 1 day 1 was observed in arm A (CC‐223 20 mg) and arm C (CC‐223 15 mg) (Figure 3). A comparison of 20‐mg and 30‐mg dose levels in arm A did not demonstrate a dose‐dependent decrease in either p4EBP1 or pAKT, a possible indication that maximal suppression of mTORC1/mTORC2 activity by CC‐223 is achieved at the 20‐mg dose level. In particular, 20 mg of CC‐223 led to median reductions in p4EBP1 and pAKT levels of approximately 30% and 70%, respectively, at 3 h post‐dose compared with baseline. In arm C, p4EBP1 levels at 3 h post‐dose increased by approximately 8% from baseline, and pAKT levels were reduced by a median of approximately 50% with 20 mg of CC‐223. When the dose of CC‐223 was reduced to 15 mg, p4EBP1 was reduced by approximately 30% and the pAKT levels remained at an approximate 50% reduction.

### 3.5 PK and pharmacodynamics

Mean plasma concentration‐time profiles and steady‐state PK parameters of CC‐223 and CC‐292 after multiple doses are shown in Figure S3. Mean plasma concentration‐versus‐time profiles are adequately characterized over the 8‐h post‐dose sampling interval. At the steady‐state, the median time to maximum plasma drug concentration (*C*_max_), *T*_max_, was 1.5 hours post‐dose, ranging from 1.0 to 2.0 h; CC‐292 T_max_ was 1.5 h, ranging from 1.0 to 3.0 h. Following attainment of *C*_max_, both CC‐223 and CC‐292 plasma concentrations declined in an approximately monophasic manner.

The mean apparent volume of distribution of CC‐223 was 127.4 L, and the mean apparent volume of distribution of CC‐292 was 334.1 L. CC‐223 had a terminal half‐life of approximately 3.6 h and apparent plasma clearance of approximately 24.3 L/h, whereas CC‐292 had a terminal half‐life of approximately 3.3 h and apparent plasma clearance of approximately 70.3 L/h. In general, as assessed from the geometric coefficient of variation, high between‐patient variability is noted for both CC‐122 area under the plasma concentration‐time curve (AUC) and C_max_, with values ranging from 105.3% to 118.1% (Figure S3). Moderate interpatient variability was observed for both CC‐292 AUC and C_max_, with values ranging from 51.3% to 56.5%. There were insufficient numbers of patients in the CC‐122‐containing arms to determine conclusively whether there were exposure‐dependent changes in Aiolos protein levels in response to CC‐122. CC‐223 exposure‐response analysis demonstrated little to no exposure‐dependent decrease in p4EBP1 and pAKT levels.

## 4 CONCLUSIONS

Despite continuing advances in the treatment of B‐cell malignancies, acquired and de novo resistance to treatment remain significant issues. Indeed, anti‐CD19 CAR T‐cell therapies represent a promising advance in the treatment of R/R DLBCL and are now the standard of care treatment for patients whose disease has progressed despite treatment with available standard therapies [42, 43, 44]. Furthermore, initial data show that CAR T‐cell therapies have promising efficacy compared with high‐dose chemotherapy and ASCT, the current standard‐of‐care second‐line treatment for DLBCL [9, 10]. It is important to note that the current study predates the introduction of CAR T‐cell therapies and other treatment options that have demonstrated durable benefits in this difficult‐to‐treat population. However, there remains a need for novel drugs and combinations so as to provide additional treatment options for those patients who experience disease progression or are not eligible for treatment with these novel regimens. This phase Ib study in patients with R/R DLBCL utilized an innovative study design to evaluate combinations of the targeted therapies CC‐122, CC‐223, and CC‐292, as doublets and as triplets with rituximab. All three targeted therapies act on pathways involved in the growth and survival of B cells, providing a rationale for the investigation of these combinations.

The CC‐122‐containing arms showed high rates of neutropenia (36.7%–45.2%), which was expected based on safety results from a previous clinical study [30]. Compared with the continuous dosing regimen used in previous studies, a five out of seven–day dosing schedule–showed improved tolerability, in terms of frequency and severity of AEs, while maintaining clinical activity [30]. The intermittent dosing regimen was designed to mitigate neutropenia by reversing an arrest in neutrophil maturation caused by depletion of Ikaros [29]. CC‐122 was poorly tolerated at doses higher than 1 mg in combination with CC‐292 (arm B). Although this combination showed evidence of antitumor activity, it also resulted in rates of grade 3/4 neutropenia comparable to those seen in other treatment arms, despite the introduction of an intermittent dosing schedule and the use of a markedly lower CC‐122 dose in arm B than in arms A and D. This observation may be explained by pharmacodynamic data showing a trend towards enhanced Aiolos degradation with CC‐122 plus CC‐292, indicating possible synergistic effects. Thus, this combination was not explored further. Also of note, the combination of CC‐223 and CC‐292 resulted in a high rate of DLTs, and both dose levels evaluated were determined to be non‐tolerated. Much like the example provided by the triplet of idelalisib, lenalidomide, and rituximab, which resulted in unexpected dose‐limiting toxicities [24], this finding indicates that any future studies of mTOR inhibitor and BTK inhibitor combinations should be done with care, particularly since the mechanisms leading to the poor tolerability seen in our study have not been elucidated.

CC‐122 plus rituximab (arm D) was considered suitable for dose expansion in patients with R/R DLBCL at the RP2D of CC‐122 3 mg QD formulated capsule for 5/7 days plus 375 mg/m^2^ rituximab. This combination was selected for further evaluation based on its preliminary antitumor activity, with an ORR of 23.3% (95% CI, 9.9–42.3), and manageable tolerability profile. The ORR with CC‐122 plus rituximab appears somewhat lower than that seen with CC‐122 plus obinutuzumab in patients with R/R DLBCL (47% [95% CI, 24–71]), although the comparability of the patient populations enrolled in these trials is unclear [31]. The dose expansion provides an opportunity to assess CC‐122 as a next‐generation cereblon‐modulating drug in the context of historical data with lenalidomide‐containing doublet regimens. In recently reported clinical studies, a novel gene expression classifier identified tumors with a high T cell and macrophage infiltrate that showed an enhanced response to CC‐122 in patients with R/R DLBCL [29, 32]. Subgroup analyses using this gene expression classifier and COO classification were investigated in the DLBCL dose‐expansion cohort, the results of which are published as a companion article in this issue of eJHaem.

Results for the CC‐122 combinations evaluated in the dose‐finding part of this study should be viewed in the context of treatment options that were approved for use in the third‐line setting after our study was conducted. As of November 2021, third‐line treatment options recommended in the National Comprehensive Cancer Network (NCCN) guidelines include anti‐CD19 CAR‐T cell therapies, in patients who have not previously received them, loncastuximab tesirine, and selinexor, as well as participation in clinical trials of novel therapies [4]. The anti‐CD19 CAR‐T cell therapies axicabtagene ciloleucel, lisocabtagene maraleucel, and tisagenlecleucel have demonstrated CR rates of 40%–58% as third‐line treatment in patients with R/R large B‐cell lymphomas, including DLBCL [42, 43, 44]. Importantly, responses to CAR T‐cell therapies have proven to be durable. The estimated 12‐month PFS rate was 65% in patients with a complete response to lisocabtagene maraleucel, 24‐month PFS was 72% in patients with a complete response to axicabtagene ciloleucel, and 36‐month PFS was 70% in patients with a complete response to tisagenlecleucel at 3 months [42, 44, 45]. In comparison, CR rates with loncastuximab tesirine and selinexor monotherapies in the third‐line setting were 24% and 12%, respectively [46, 47].

Several doublet and triplet combinations have been evaluated in patients who have received two or more prior therapies. Treatment with the ADC polatuzumab vedotin in combination with bendamustine and rituximab (pola‐BR) resulted in a significantly higher CR rate and reduced the risk of death by 58% compared with BR alone in patients with transplantation‐ineligible R/R DLBCL [48]. The combination of the CD19 mAb tafasitamab plus lenalidomide produced an ORR of 60% with CRs observed in 43% of patients with R/R DLBCL ineligible for ASCT [17]. These regimens are recommended as second‐line or later therapy in the NCCN guidelines [4]. Clinical activity was also observed in a study of ibrutinib plus lenalidomide in DLBCL, with an ORR of 38% and median DOR of 15.9 months; of particular interest, increased ORR was observed in non‐GCB‐like DLBCL [19]. These studies indicate not only that novel triplet therapies are feasible, but that toxicity management and patient selection are key to identifying combination doses that may lead to transformational efficacy in specific patient populations.

In conclusion, this dose‐escalation study evaluated multiple combinations of three novel therapies, as doublets and as triplets with rituximab, in heavily pretreated patients with R/R DLBCL. The therapies were selected for investigation based on preclinical studies and knowledge of tumor biology that suggested their combination would result in an additional benefit, as well as the results of previous clinical studies demonstrating antitumor activity of combinations containing drugs with similar mechanisms of actions. Investigation of pharmacodynamic markers was used to inform alterations to the CC‐122 dosing schedule with the goal of improving tolerability, as well as the selection of combinations for further investigation through identification of potential synergies with deleterious effects. Of the regimens examined in this study, the combination of CC‐122 and rituximab was well tolerated and demonstrated preliminary efficacy in patients with R/R DLBCL. The efficacy of this combination in terms of ORR and DOR, as well as the antitumor activity of CC‐122 in patients who have previously received lenalidomide, has been evaluated in patients with DLBCL and FL in the part B dose expansion. Results from the dose‐expansion part are reported in a separate manuscript in this issue of eJHaem.

## FUNDING INFORMATION

This work was funded was provided by Celgene, a Bristol Myers Squibb company.

## CONFLICT OF INTEREST

Vincent Ribrag received honoraria from Gilead, Infinity, ArgenX, Merck Sharp & Dohme, Bristol Myers Squibb, Epizyme, Nanostring, Incyte, Roche, and AstraZeneca; served as a consultant or advisor for Servier; and received research funding from ArgenX. Julio C. Chavez served as a consultant or advisor for Novartis, Celgene, a Bristol‐Myers Squibb Company, Bayer, Morphosys, Karyopharm, AstraZeneca, Verastem, Pfizer, and Genentech; received research funding from Merck; and served on the speaker's bureau for Genentech and AstraZeneca. Jason Kaplan served as a consultant or advisor for and received research funding from Seattle Genetics, and received travel funding from Curis. Jason C. Chandler served as a consultant or advisor for Janssen and Axess Oncology. Armando Santoro served as a consultant or advisor for ArQule, Sanofi, Bristol Myers Squibb, Servier, Gilead, Pfizer, Eisai, Bayer, and Merck Sharpe and Dohme; and served on the speaker's bureau for Takeda, Bristol Myers Squibb, Roche, AbbVie, Amgen, Celgene, a Bristol‐Myers Squibb company, Servier, Gilead, AstraZeneca, Pfizer, ArQule, Lilly, Sandoz, Eisai, Novartis, Bayer, and Merck Sharpe and Dohme. Paolo Corradini received honoraria from Janssen, Gilead, AbbVie, Takeda, Roche, Novartis, and Celgene, a Bristol‐Myers Squibb Company; served as a consultant or advisor for Novartis, Janssen, Celgene, a Bristol‐Myers Squibb company, and Gilead; received travel funding from Novartis, AbbVie, and Gilead; and served on the speaker's bureau for Novartis. Ian W. Flinn received research funding from AbbVie, Acerta, Agios, ArQule, BeiGene, Calithera, Celgene, a Bristol‐Myers Squibb Company, Constellation, Curis, Forma, Forty‐Seven Inc, Genentech, Gilead, Incyte, Infinity, Janssen, Karyopharm, Kite, Merck, Novartis, Pfizer, Portola, Roche, Seattle Genetics, Takeda, Teva, TG Therapeutics, Trillium, and Verastem. Ranjana Advani served as a consultant or advisor for AstraZeneca, Bayer Healthcare Pharmaceuticals, Cell Medica, Genentech/Roche, Gilead Kite Pharma, Kyowa, Seattle Genetics, and Takeda; and received research funding from Agensys, Celgene, a Bristol‐Myers Squibb company, Forty‐Seven Inc., Genentech/Roche, Janssen Pharmaceutical, Kura, Merck, Millenium, Pharmacyclics, Regeneron, and Seattle Genetics. Philippe A. Cassier received honoraria from Amgen, AstraZeneca, Blueprint Medicines, Novartis, and Roche/Genentech; received research funding from AbbVie, AstraZeneca, Bayer, Blueprint Medicines, Bristol Myers Squibb, Celgene, a Bristol‐Myers Squibb Company, GlaxoSmithKline, Innate Pharma, Janssen, Lilly, Loxo, Merck Serono, Merck Sharp & Dohme, Novartis, Plexxikon, Roche/Genentech, Taiho Pharmaceutical, Toray Industries, and Transgene; and received travel funding from Amgen, Bristol Myers Squibb, Merck Sharp & Dohme, Netris Pharma, Novartis, and Roche. Randeep Sangha received honoraria from Pfizer, Boehringer Ingelheim, AstraZeneca, Roche, Lundbeck, Bristol Myers Squibb, Merck, AbbVie, and Takeda; served as a consultant or advisor for Boehringer Ingelheim, Roche, Lundbeck, Bristol Myers Squibb, Merck, AbbVie, Takeda, and Teva; and received research funding from Bristol Myers Squibb, AbbVie, Takeda, Pharmacyclics, Morphosys, Roche, Merck, Celgene, a Bristol‐Myers Squibb company, and Novartis. Iris Isufi served as a consultant or advisor for AstraZeneca, Celgene, a Bristol‐Myers Squibb company/Jazz Pharmaceuticals, and Kite Pharma; and received travel funding from Celgene, a Bristol‐Myers Squibb company, and Kite Pharma. Shailaja Uttamsingh, Patrick R. Hagner, Anita K. Gandhi, and Michael Pourdehnad are employed by and have equity ownership with Bristol Myers Squibb. Harald Haeske has equity ownership with Pieris AG and served as a consultant or advisor for Celgene, a Bristol‐Myers Squibb company, and 4SC AG. Kristen Hege is employed by and holds patents with Bristol Myers Squibb; has equity ownership with Bristol Myers Squibb, Arcus Biosciences, and Mersana Therapeutics; and served on the Board of Directors for Arcus Biosciences, Mersana Therapeutics, and the Society for Immunotherapy of Cancer. John Kuruvilla received honoraria from Amgen, AstraZeneca, Bristol Myers Squibb, Celgene, a Bristol‐Myers Squibb Company, Gilead, Janssen, Karyopharm Therapeutics, Merck, Novartis, Roche, and Seattle Genetics; and served as a consultant or advisor for AbbVie, Bristol Myers Squibb, Gilead Sciences, Karyopharm Therapeutics, Merck, Roche, and Seattle Genetics; and received research funding from Janssen and Roche. All other authors declare no conflict of interest.

## AUTHOR CONTRIBUTIONS

**Vincent Ribrag**: Data curation, formal analysis, investigation, methodology, writing‐original draft, project administration, writing‐review, and editing. **Julio C. Chavez**: Data curation, formal analysis, investigation, methodology, writing‐original draft, project administration, writing‐review, and editing. **Carola Boccomini**: Data curation, formal analysis, investigation, methodology, writing‐original draft, project administration, writing‐review, and editing. **Jason Kaplan**: Data curation, formal analysis, investigation, methodology, writing‐original draft, project administration, writing‐review, and editing. **Jason C. Chandler**: Data curation, formal analysis, investigation, methodology, writing‐original draft, project administration, writing‐review, and editing. **Armando Santoro**: Data curation, formal analysis, investigation, methodology, writing‐original draft, project administration, writing‐review, and editing. **Paolo Corradini**: Data curation, formal analysis, investigation, methodology, writing‐original draft, project administration, writing‐review, and editing. **Ian W. Flinn**: Data curation, formal analysis, investigation, methodology, writing‐original draft, project administration, writing‐review, and editing. **Ranjana Advani**: Data curation, formal analysis, investigation, methodology, writing‐original draft, project administration, writing‐review, and editing. **Philippe A. Cassier**: Data curation, formal analysis, investigation, methodology, writing‐original draft, project administration, writing‐review, and editing. **Randeep Sangha**: Data curation, formal analysis, investigation, methodology, writing‐original draft, project administration, writing‐review, and editing. **Vaishalee P. Kenkre**: Data curation, formal analysis, investigation, methodology, writing‐original draft, project administration, writing‐review, and editing. **Iris Isufi**: Data curation, formal analysis, investigation, methodology, writing‐original draft, project administration, writing‐review, and editing. **Shailaja Uttamsingh**: Data curation, formal analysis, investigation, methodology, writing‐original draft, project administration, writing‐review, and editing. **Patrick R. Hagner**: Data curation, formal analysis, investigation, methodology, writing‐original draft, project administration, writing‐review, and editing. **Anita K. Gandhi**: Data curation, formal analysis, investigation, methodology, writing‐original draft, project administration, writing‐review, and editing. **Frank Shen**: Data curation, formal analysis, investigation, methodology, writing‐original draft, project administration, writing‐review, and editing. **Sophie Michelliza**: Data curation, formal analysis, investigation, methodology, writing‐original draft, project administration, writing‐review, and editing. **Harald Haeske**: Data curation, formal analysis, investigation, methodology, writing‐original draft, project administration, writing‐review, and editing. **Kristen Hege**: Conceptualization, data curation, formal analysis, investigation, methodology, writing‐original draft, project administration, writing‐review, and editing. **Michael Pourdehnad**: Conceptualization, data curation, formal analysis, investigation, methodology, writing‐original draft, project administration, writing‐review, and editing. **John Kuruvilla**: Data curation, formal analysis, investigation, methodology, writing‐original draft, project administration, writing‐review, and editing.

## Supporting information



SUPPORTING INFORMATION

## References

[1] Armitage JO , Weisenburger DD . New approach to classifying non‐Hodgkin's lymphomas: clinical features of the major histologic subtypes. Non‐Hodgkin's Lymphoma Classification Project. J Clin Oncol. 1998;16:2780–95. 9704731 10.1200/JCO.1998.16.8.2780

[2] Armitage JO , Gascoyne RD , Lunning MA , Cavalli F . Non‐Hodgkin lymphoma. Lancet 2017;390:298–310. 28153383 10.1016/S0140-6736(16)32407-2

[3] Galaznik A , Huelin R , Stokes M , Guo Y , Hoog M , Bhagnani T , et al. Systematic review of therapy used in relapsed or refractory diffuse large B‐cell lymphoma and follicular lymphoma. Future Sci OA. 2018;4:FSO322. 30112190 10.4155/fsoa-2018-0049 PMC6088264

[4] National Comprehensive Cancer Network . NCCN clinical practice guidelines in oncology (NCCN guidelines) B‐cell lymphomas, version 5. 2021. https://www.nccn.org/professionals/physician_gls/pdf/b‐cell.pdf. Accessed November 17, 2021.

[5] Crump M , Neelapu SS , Farooq U , Van Den Neste E , Kuruvilla J , Westin J , et al. Outcomes in refractory diffuse large B‐cell lymphoma: results from the international SCHOLAR‐1 study. Blood 2017;130:1800–8. 28774879 10.1182/blood-2017-03-769620 PMC5649550

[6] Hitz F , Connors JM , Gascoyne RD , Hoskins P , Moccia A , Savage KJ , et al. Outcome of patients with primary refractory diffuse large B cell lymphoma after R‐CHOP treatment. Ann Hematol. 2015;94:1839–43. 26246466 10.1007/s00277-015-2467-z

[7] Van Den Neste E , Schmitz N , Mounier N , Gill D , Linch D , Trneny M , et al. Outcome of patients with relapsed diffuse large B‐cell lymphoma who fail second‐line salvage regimens in the International CORAL study. Bone Marrow Transplant. 2016;51:51–57. 26367239 10.1038/bmt.2015.213

[8] Nagle SJ , Woo K , Schuster SJ , Nasta SD , Stadtmauer E , Mick R , et al. Outcomes of patients with relapsed/refractory diffuse large B‐cell lymphoma with progression of lymphoma after autologous stem cell transplantation in the rituximab era. Am J Hematol. 2013;88:890–4. 23813874 10.1002/ajh.23524

[9] Bristol Myers Squibb . Bristol Myers Squibb announces positive topline results from phase 3 TRANSFORM trial evaluating breyanzi (lisocabtagene maraleucel) versus chemotherapy followed by stem cell transplant in second‐line relapsed or refractory large B‐cell lymphoma. 2021. Available from: https://www.bloomberg.com/press-releases/2021-06-10/bristol-myers-squibb-announces-positive-topline-results-from-phase-3-transform-trial-evaluating-breyanzi-lisocabtagene

[10] Kite . Kite Announces Yescarta CAR T‐cell therapy improved event‐free survival by 60% over chemotherapy plus stem cell transplant in second‐line relapsed or refractory large B‐cell lymphoma. 2021. Available from: https://www.gilead.com/news-and-press/press-room/press-releases/2021/6/kite-announces-yescarta-car-tcell-therapy-improved-eventfree-survival-by-60-over-chemotherapy-plus-stem-cell-transplant-in-secondline-relapsed-or

[11] Czuczman MS , Trneny M , Davies A , Rule S , Linton KM , Wagner‐Johnston N , et al. A phase 2/3 multicenter, randomized, open‐label study to compare the efficacy and safety of lenalidomide versus investigator's choice in patients with relapsed or refractory diffuse large B‐Cell lymphoma. Clin Cancer Res. 2017;23:4127–37. 28381416 10.1158/1078-0432.CCR-16-2818 PMC8171498

[12] Czuczman MS , Vose J , Zinzani PL , Reeder CB , Tuscano JM , Lossos IS , et al. Lenalidomide monotherapy is clinically active in patients with relapsed/refractory diffuse large B‐cell lymphoma (DLBCL): a pooled analysis of data from 2 Phase II studies (NHL‐002/003). Haematologica 2010;95(Suppl 2):0574.

[13] Wilson WH , Young RM , Schmitz R , Yang Y , Pittaluga S , Wright G , et al. Targeting B cell receptor signaling with ibrutinib in diffuse large B cell lymphoma. Nat Med. 2015;21:922–6. 26193343 10.1038/nm.3884 PMC8372245

[14] Smith SM , van Besien K , Karrison T , Dancey J , McLaughlin P , Younes A , et al. Temsirolimus has activity in non‐mantle cell non‐Hodgkin's lymphoma subtypes: the University of Chicago phase II consortium. J Clin Oncol. 2010;28:4740–6. 20837940 10.1200/JCO.2010.29.2813 PMC3020703

[15] Wang L , Li L‐r , Young KH . New agents and regimens for diffuse large B cell lymphoma. J Hematol Oncol. 2020;13:175. 33317571 10.1186/s13045-020-01011-z PMC7734862

[16] Leonard JP , Jung SH , Johnson J , Pitcher BN , Bartlett NL , Blum KA , et al. Randomized trial of lenalidomide alone versus lenalidomide plus rituximab in patients with recurrent follicular lymphoma: CALGB 50401 (Alliance). J Clin Oncol. 2015;33:3635–40. 26304886 10.1200/JCO.2014.59.9258 PMC4622102

[17] Salles G , Duell J , González Barca E , Tournilhac O , Jurczak W , Liberati AM , et al. Tafasitamab plus lenalidomide in relapsed or refractory diffuse large B‐cell lymphoma (L‐MIND): a multicentre, prospective, single‐arm, phase 2 study. Lancet Oncol. 2020;21:978–88. 32511983 10.1016/S1470-2045(20)30225-4

[18] Witzig TE , LaPlant B , Habermann TM , McPhail E , Inwards DJ , Micallef IN , et al. High rate of event‐free survival at 24 months with everolimus/RCHOP for untreated diffuse large B‐cell lymphoma: updated results from NCCTG N1085 (Alliance). Blood Cancer J. 2017;7:e576. 28649983 10.1038/bcj.2017.57 PMC5520404

[19] Goy A , Ramchandren R , Ghosh N , Munoz J , Morgan DS , Dang NH , et al. Ibrutinib plus lenalidomide and rituximab has promising activity in relapsed/refractory non–germinal center B‐cell‐like DLBCL. Blood 2019;134:1024–36. 31331917 10.1182/blood.2018891598 PMC6764267

[20] Young RM , Phelan JD , Wilson WH , Staudt LM . Pathogenic B‐cell receptor signaling in lymphoid malignancies: new insights to improve treatment. Immunol Rev. 2019;291:190–213. 31402495 10.1111/imr.12792 PMC6693651

[21] Nowakowski GS , Czuczman MS . ABC, GCB, and double‐hit diffuse large B‐Cell lymphoma: does subtype make a difference in therapy selection? Am Soc Clin Oncol Educ Book. 2015:e449–57. 25993209 10.14694/EdBook_AM.2015.35.e449

[22] Nowakowski GS , Hong F , Scott DW , Macon WR , King RL , Habermann TM , et al. Addition of lenalidomide to R‐CHOP improves outcomes in newly diagnosed diffuse large B‐Cell lymphoma in a randomized phase II US intergroup study ECOG‐ACRIN E1412. J Clin Oncol. 2021;39:1329–38. 33555941 10.1200/JCO.20.01375 PMC8078264

[23] Boshuizen J , Peeper DS . Rational cancer treatment combinations: an urgent clinical need. Mol Cell. 2020;78:1002–18. 32559422 10.1016/j.molcel.2020.05.031

[24] Smith SM , Pitcher BN , Jung SH , Bartlett NL , Wagner‐Johnston N , Park SI , et al. Safety and tolerability of idelalisib, lenalidomide, and rituximab in relapsed and refractory lymphoma: the alliance for clinical trials in oncology A051201 and A051202 phase 1 trials. Lancet Haematol. 2017;4:e176–82. 28314699 10.1016/S2352-3026(17)30028-5 PMC5499150

[25] Hagner PR , Man HW , Fontanillo C , Wang M , Couto S , Breider M , et al. CC‐122, a pleiotropic pathway modifier, mimics an interferon response and has antitumor activity in DLBCL. Blood 2015;126:779–89. 26002965 10.1182/blood-2015-02-628669 PMC4528065

[26] Hagner P , Wang M , Couto S , Breider M , Fontanillo C , Trotter M , et al. CC‐122 has potent anti‐lymphoma activity through destruction of the Aiolos and Ikaros transcription factors and induction of interferon response pathways. Blood 2014;124:3035.25395139

[27] Gandhi AK , Vincent R , Carpio C , Stoppa AM , Gharibo MM , Damian S , et al. CC‐122 expands activated and memory CD4 and CD8 T cells in vivo and induces T Cell activation ex vivo in subjects with relapsed or refractory diffuse large B cell lymphoma and multiple myeloma. Blood 2015;126:2704.26337492

[28] Cubillos‐Zapata C , Cordoba R , Avendano‐Ortiz J , Arribas‐Jiménez C , Hernández‐Jiménez E , Toledano V , et al. CC‐122 immunomodulatory effects in refractory patients with diffuse large B‐cell lymphoma. Oncoimmunology. 2016;5:e1231290. 28255524 10.1080/2162402X.2016.1231290 PMC5325046

[29] Carpio C , Bouabdallah R , Ysebaert L , Sancho JM , Salles G , Cordoba R , et al. Avadomide monotherapy in relapsed/refractory DLBCL: safety, efficacy, and a predictive gene classifier. Blood 2020;135:996–1007. 31977002 10.1182/blood.2019002395 PMC7099331

[30] Rasco DW , Papadopoulos KP , Pourdehnad M , Gandhi AK , Hagner PR , Li Y , et al. A first‐in‐human study of novel cereblon modulator avadomide (CC‐122) in advanced malignancies. Clin Cancer Res. 2019;25:90–98. 30201761 10.1158/1078-0432.CCR-18-1203

[31] Michot JM , Bouabdallah R , Vitolo U , Doorduijn JK , Salles G , Chiappella A , et al. Avadomide plus obinutuzumab in patients with relapsed or refractory B‐cell non‐Hodgkin lymphoma (CC‐122‐NHL‐001): a multicentre, dose escalation and expansion phase 1 study. Lancet Haematol. 2020;7:e649–59. 32758434 10.1016/S2352-3026(20)30208-8

[32] Risueño A , Hagner PR , Towfic F , Fontanillo C , Djebbari A , Parker JS , et al. Leveraging gene expression subgroups to classify DLBCL patients and select for clinical benefit from a novel agent. Blood 2020;135:1008–18. 31977005 10.1182/blood.2019002414 PMC7099333

[33] Bendell JC , Kelley RK , Shih KC , Grabowsky JA , Bergsland E , Jones S , et al. A phase I dose‐escalation study to assess safety, tolerability, pharmacokinetics, and preliminary efficacy of the dual mTORC1/mTORC2 kinase inhibitor CC‐223 in patients with advanced solid tumors or multiple myeloma. Cancer 2015;121:3481–90. 26177599 10.1002/cncr.29422 PMC4832308

[34] Mortensen DS , Fultz KE , Xu S , Xu W , Packard G , Khambatta G , et al. CC‐223, a potent and selective inhibitor of mTOR kinase: in vitro and in vivo characterization. Mol Cancer Ther. 2015;14:1295–305. 25855786 10.1158/1535-7163.MCT-14-1052

[35] Xie Z , Wang J , Liu M , Chen D , Qiu C , Sun K . CC‐223 blocks mTORC1/C2 activation and inhibits human hepatocellular carcinoma cells in vitro and in vivo. PLoS One. 2017;12:e0173252. 28334043 10.1371/journal.pone.0173252 PMC5363890

[36] Brown JR , Harb WA , Hill BT , Gabrilove J , Sharman JP , Schreeder MT , et al. Phase I study of single‐agent CC‐292, a highly selective Bruton's tyrosine kinase inhibitor, in relapsed/refractory chronic lymphocytic leukemia. Haematologica 2016;101:e295–8. 27151992 10.3324/haematol.2015.140806 PMC5004476

[37] Barnes JA , Jacobsen E , Feng Y , Freedman A , Hochberg EP , LaCasce AS , et al. Everolimus in combination with rituximab induces complete responses in heavily pretreated diffuse large B‐cell lymphoma. Haematologica 2013;98:615–9. 23144193 10.3324/haematol.2012.075184 PMC3659994

[38] Wang M , Fowler N , Wagner‐Bartak N , Feng L , Romaguera J , Neelapu SS , et al. Oral lenalidomide with rituximab in relapsed or refractory diffuse large cell, follicular and transformed lymphoma: a phase II clinical trial. Leukemia 2013;27:1902–9. 23545991 10.1038/leu.2013.95

[39] Reeder CB , Ansell SM . Novel therapeutic agents for B‐cell lymphoma: developing rational combinations. Blood 2011;117:1453–62. 20978267 10.1182/blood-2010-06-255067

[40] Storer BE . Design and analysis of phase I clinical trials. Biometrics 1989;45:925–37. 2790129

[41] Cheson BD , Pfistner B , Juweid ME , Gascoyne RD , Specht L , Horning SJ , et al. Revised response criteria for malignant lymphoma. J Clin Oncol. 2007;25:579–86. 17242396 10.1200/JCO.2006.09.2403

[42] Locke FL , Ghobadi A , Jacobson CA , Miklos DB , Lekakis LJ , Oluwole OO , et al. Long‐term safety and activity of axicabtagene ciloleucel in refractory large B‐cell lymphoma (ZUMA‐1): a single‐arm, multicentre, phase 1–2 trial. Lancet Oncol. 2019;20:31–42. 30518502 10.1016/S1470-2045(18)30864-7 PMC6733402

[43] Schuster SJ , Bishop MR , Tam CS , Waller EK , Borchmann P , McGuirk JP , et al. Tisagenlecleucel in adult relapsed or refractory diffuse large B‐cell lymphoma. N Engl J Med. 2019;380:45–56. 30501490 10.1056/NEJMoa1804980

[44] Abramson JS , Palomba ML , Gordon LI , Lunning MA , Wang M , Arnason J , et al. Lisocabtagene maraleucel for patients with relapsed or refractory large B‐cell lymphomas (TRANSCEND NHL 001): a multicentre seamless design study. Lancet 2020;396:839–52. 32888407 10.1016/S0140-6736(20)31366-0

[45] Schuster SJ , Tam CS , Borchmann P , Worel N , McGuirk JP , Holte H , et al. Long‐term clinical outcomes of tisagenlecleucel in patients with relapsed or refractory aggressive B‐cell lymphomas (JULIET): a multicentre, open‐label, single‐arm, phase 2 study. Lancet Oncol. 2021;22:1403–15. 34516954 10.1016/S1470-2045(21)00375-2

[46] Caimi PF , Ai W , Alderuccio JP , Ardeshna KM , Hamadani M , Hess B , et al. Loncastuximab tesirine in relapsed or refractory diffuse large B‐cell lymphoma (LOTIS‐2): a multicentre, open‐label, single‐arm, phase 2 trial. Lancet Oncol. 2021;22:790–800. 33989558 10.1016/S1470-2045(21)00139-X

[47] Kalakonda N , Maerevoet M , Cavallo F , Follows G , Goy A , Vermaat JSP , et al. Selinexor in patients with relapsed or refractory diffuse large B‐cell lymphoma (SADAL): a single‐arm, multinational, multicentre, open‐label, phase 2 trial. Lancet Haematol. 2020;7:e511–22. 32589977 10.1016/S2352-3026(20)30120-4

[48] Sehn LH , Herrera AF , Flowers CR , Kamdar MK , McMillan A , Hertzberg M , et al. Polatuzumab vedotin in relapsed or refractory diffuse large B‐cell lymphoma. J Clin Oncol. 2020;38:155–65. 31693429 10.1200/JCO.19.00172 PMC7032881

