# Supplementary material for: Phase Ib study of combinations of avadomide (CC‐122), CC‐223, CC‐292, and rituximab in patients with relapsed/refractory diffuse large B‐cell lymphoma
Source: EJHaem. 2022 Jan 14;3(1):139–53. doi: 10.1002/jha2.375 (PMC9176062; doi:10.1002/jha2.375)

# Supplementary materials

## Supplementary Methods

### Measurement of antibody-dependent, cell-mediated cytotoxicity

Purified natural killer (NK) cells from patient peripheral blood mononuclear cell samples were treated with 10 ng/mL rh-IL-2 (R&D) alone or with avadomide (1–100 nM) for 24 hours at 37°C. Cells (1 × 10^6^ cells/mL) from 2 FL cell lines, DOHH2 and RL, were stained with CellTrace carboxyfluorescein succinimidyl ester (CFSE; Thermo Fisher Scientific) (200 nmol/L) for 5 minutes at room temperature, then treated with dimethyl sulfoxide (DMSO; vehicle) or rituximab (1 μg/mL) for 1 hour prior to coculture. Target tumor DOHH2 and RL cells (2 × 10^4^ cells) were added to DMSO or avadomide pretreated effector NK cells at a 1:5 ratio (target to effector cells) and were incubated for 4 hours at 37°C. Cells were stained with Annexin V-PE and TO-PRO-3 (Thermo Fisher Scientific) according to the manufacturer’s instructions. The percentage of tumor apoptosis was determined by sequential gating for CFSE-positive and double-positive (Annexin V-PE and TO-PRO-3–positive) cells.

### DOHH2 xenograft model

Female severe combined immunodeficiency (SCID) mice (CB17/Icr-*Prkdcscid*, Charles River) were 8 weeks old, with body weights ranging from 15.0 g to 23.2 g on day 1 of these studies. Each SCID mouse was injected subcutaneously in the right flank with 5 × 10^6^ DOHH2 cells (0.2-mL cell suspension). Tumors were calipered in 2 dimensions to monitor growth as their mean volume approached 100 to 150 mm^3^. Fourteen days after tumor cell implantation, mice were sorted into treatment groups of 10 mice per group. Tumors were calipered twice weekly during the study. Avadomide was suspended in 0.5% carboxymethyl cellulose (0.25% Tween-80 in deionized water). Vehicle control and avadomide were each administered via oral gavage once daily; rituximab (1 mg/kg) was administered once weekly for the duration of the study.

### Patients

Eligible patients had histologically or cytologically confirmed relapsed or refractory DLBCL, including transformed indolent lymphoma. Pathology was determined by local assessment. All patients were chemorefractory. Patients with CD20-positive tumors by local assessment were required to have received an anti-CD20 monoclonal antibody therapy; all patients must have received an anthracycline-containing chemotherapy and ≥1 prior salvage treatment (unless ineligible for autologous stem cell transplant [ASCT]). Patients were also required to have documented proof of ineligibility for ASCT at the time of enrollment, with ineligibility defined as follows: having a condition, including laboratory abnormalities or clinical symptoms, that place the patient at an unacceptable risk if he/she underwent ASCT; patient declining ASCT; physician determined the patient’s disease could not be adequately treated by ASCT for various reasons, such as active disease following salvage therapy; insufficient CD34 stem cell collection.

Additional inclusion criteria include measurable disease >1.5 cm in the long axis or >1.0 cm in both long and short axes, an Eastern Cooperative Oncology Group performance status score of 0 or 1, adequate hematologic function (absolute neutrophil count ≥1.5 × 10^9^/L without growth factor support for 7 days [14 days if pegfilgrastim was administered], hemoglobin ≥8 g/dL, platelets ≥100 ×10^9^/L without transfusion for 7 days), hepatic function (serum bilirubin ≤1.5 × upper limit of normal [ULN], alanine aminotransferase and aspartate aminotransferase ≤ 2.5 × ULN or 5.0 × ULN if liver tumor was present), renal function (serum creatinine 24-hour clearance ≥50 mL/min), and potassium levels within normal limits or correctable with supplements. Exclusion criteria include symptomatic central nervous system involvement; known acute or chronic pancreatitis or diabetes on active treatment; peripheral neuropathy grade ≥2 per National Cancer Institute Common Terminology Criteria for AEs (NCI CTCAE), version 4.03; persistent diarrhea or malabsorption grade ≥2 despite optimal medical management; impaired cardiac function or clinically significant cardiac diseases; diabetes, fasting blood glucose ≥126 mg/dL, or hemoglobin A1c ≥6.5% in patients treated in the CC-223 arm; prior ASCT ≤3 months before first dose or with standard or reduced-intensity conditioning; prior systemic anticancer treatments ≤5 half-lives or 4 weeks before the start of study drug, whichever was shorter; prior treatment with a dual mammalian target of rapamycin complex 1/mammalian target of rapamycin complex 2 inhibitor in the CC‑223 arms or Bruton tyrosine kinase inhibitors in the CC-292 arms; treatment-related myelodysplastic syndrome or a history of concurrent secondary cancers requiring active, ongoing treatment; chronic use of proton pump inhibitors or H_2_ antagonists within 7 days of first dose in CC-292 arms; known HIV infection, or known chronic active hepatitis B or C virus infection; or any other significant medical condition that would result unacceptably high risk.

### Study assessments

DLTs were defined as follows:

- Any grade 4 nonhematologic toxicity of any duration during cycle 1 that was suspected to be related to study treatment
- Grade 3 bilirubin elevation, whether symptomatic or asymptomatic, and other hepatic laboratory abnormalities, including the following:
  - Elevated bilirubin at screening due to Gilbert’s syndrome or hemolysis if total bilirubin increased to twice the highest study-recorded pretreatment value or met grade 3 criteria
  - Transaminase elevation ≥3 times the ULN
  - Total bilirubin more than twice the highest study-recorded pretreatment value in the absence of other medical reasons or concomitant medication
- Clinically relevant nonhematologic toxicity that was suspected to be related to study drugs and that occurred during cycle 1 and was grade 3 with the following exceptions:
  - Grade 3 acneiform, pustular, or maculopapular rash that resolved to grade ≤2 within 7 days of dose interruption and did not recur at the same level upon resumption of study treatment at the same dose level (with medical management)
  - Grade 3 diarrhea or vomiting lasting <3 days (with medical management)
  - Grade 3 fatigue or oral mucositis/stomatitis that resolved to grade ≤2 within 7 days of dose interruption and did not recur at the same level upon resumption study treatment at the same dose level (with medical management)
  - Tumor lysis syndrome that did not progress to grade 4 and resolved within 7 days with medical management
  - A nonhematologic grade 3 clinical laboratory adverse event that was asymptomatic and rapidly reversible (ie, returned to baseline or grade ≤1 within 7 days)
- Hyperglycemia meeting the following criteria:
  - Grade 2 fasting hyperglycemia lasting >14 days
  - Grade ≥3 hyperglycemia lasting >4 days
  - Grade 4 hyperglycemia lasting ≥12 hours despite optimal medical treatment
  - Hyperglycemia associated with diabetic ketoacidosis or nonketotic hyperosmolar coma regardless of glucose level
- Hematological toxicities as follows:
  - Any febrile neutropenia
  - Grade 4 neutropenia lasting >7 days
  - Grade 4 thrombocytopenia lasting >24 hours
  - Grade 3 or 4 thrombocytopenia with clinically significant bleeding
- Any AE suspected to be treatment-related and necessitating dose reduction during cycle 1.

**TABLE S1** Dose levels and treatment arms

| **Arm A** |  | | |
| --- | --- | --- | --- |
| **Dose level** | **Avadomide  (mg QD)** | **CC-223  (mg QD)** | **Rituximab  (mg/m^2^ q28)** |
| 1 | 2 | 20 | - |
| 2 | 2 | 30 | - |
| 3 | 2 | 20 | 375 |
| 4^a^ | 3 (5/7 d) | 20 | 375 |
| 5^a^ | 4 (5/7 d) | 20 | 375 |
| **Arm B** |  | | |
| **Dose level** | **Avadomide  (mg QD)** | **CC-292  (mg BID)** | **Rituximab  (mg/m^2^ q28)** |
| 1 | 1 | 500 | - |
| 2 | 2 | 500 | - |
| 3^a^ | 1 (5/7 d) | 500 | - |
| 4^a^ | 1 (5/7 d) | 500 | 375 |
| 5^a^ | 2 (5/7 d) | 500 | 375 |
| **Arm C** |  | | |
| **Dose level** | **CC-223  (mg QD)** | **CC-292  (mg BID)** |  |
| 1 | 15 | 500 |  |
| 2 | 20 | 500 |  |
| **Arm D** |  | | |
| **Dose level** | **Avadomide  (mg QD)** | **Rituximab  (mg/m^2^ q28)** |  |
| 1 | 2 | 375 |  |
| 2^a^ | 2 (5/7 d) | 375 |  |
| 3^a^ | 3 (5/7 d) | 375 |  |
| 4^a^ | 4 (5/7 d) | 375 |  |
| 5^a^ | 3 (5/7 d; formulated capsule) | 375 |  |

Abbreviations: 5/7 d, 5 consecutive days out of 7; BID, twice daily; q28, once per cycle; QD, once daily.

^a^All avadomide-containing arms dose escalated to establish maximum-tolerated dose after switching to intermittent schedule (5/7 d).

TABLE S2 Treatment duration, modification, and exposure (safety population)

|  | **Arm A** | **Arm B** | **Arm C** | **Arm D** |  |
| --- | --- | --- | --- | --- | --- |
|  | **Avadomide + CC-223 ± R  (n = 31)** | **Avadomide + CC-292 ± R  (n = 27)** | **CC-292  + CC-223**  **(n = 14)** | **Avadomide + R  (n = 30)** | **Overall** |
| Median overall treatment duration (range), days^a^ | 84.0 (12-960) | 57.0 (12-803) | - | 38.5 (7-233) | - |
| **Avadomide** | **n = 31** | **n = 27** | **-** | **n = 30** | **n = 88** |
| Median duration (range), days^a^ | 72.0 (12-941) | 56.0 (10-765) | - | 47.5 (7-605) | 56.0 (7-941) |
| Median no. of cycles (range) | 2 (0-33) | 2 (0-24) | - | 1 (0-21) | 2 (0-33) |
| Median relative dose intensity (range)^b^ | 1.0 (0.7-1.0) | 1.0 (0.6-1.0) | - | 1.0 (0.5-1.0) | 1.0 (0.5-1.0) |
| ≥1 dose reduction, n (%) | 10 (32.3) | 3 (11.1) | - | 5 (16.7) | 18 (20.5) |
| Adverse event | 6 (19.4) | 2 (7.4) | - | 3 (10.0) | 11 (12.5) |
| Per protocol | 0 | 0 | - | 1 (3.3) | 1 (1.1) |
| Other | 3 (9.7) | 2 (7.4) | - | 0 | 5 (5.7) |
| Missing | 3 (9.7) | 1 (3.7) | - | 1 (3.3) | 5 (5.7) |
| ≥1 dose interruption, n (%) | 25 (80.6) | 17 (63.0) | - | 17 (56.7) | 59 (67.0) |
| Adverse event | 23 (74.2) | 15 (55.6) | - | 11 (36.7) | 49 (55.7) |
| Per protocol | 2 (6.5) | 1 (3.7) | - | 3 (10.0) | 6 (6.8) |
| Other | 13 (41.9) | 12 (44.4) | - | 10 (33.3) | 35 (39.8) |
| Missing | 3 (9.7) | 1 (3.7) | - | 1 (3.3) | 5 (5.7) |
| **CC-223** | **n = 31** |  | **n = 14** |  | **n = 45** |
| Median duration (range), days^a^ | 68.0 (12-937) | - | 35.5 (7-112) | - | 56.0 (7-937) |
| Median no. of cycles (range) | 2 (0-33) | - | 1 (0-3) | - | 2 (0-33) |
| Median relative dose intensity (range)^b^ | 1.0 (0.7-1.0) | - | 1.0 (0.6-1.0) | - | 1.0 (0.6-1.0) |
| ≥1 dose reduction, n (%) | 10 (32.3) | - | 1 (7.1) | - | 11 (24.4) |
| Adverse event | 7 (22.6) | - | 1 (7.1) | - | 8 (17.8) |
| Per protocol | 0 | - | 0 | - | 0 |
| Other | 2 (6.5) | - | 0 | - | 2 (4.4) |
| Missing | 1 (3.2) | - | 0 | - | 1 (2.2) |
| ≥1 dose interruption, n (%) | 25 (80.6) | - | 9 (64.3) | - | 34 (75.6) |
| Adverse event | 23 (74.2) | - | 8 (57.1) | - | 31 (68.9) |
| Per protocol | 1 (3.2) | - | 1 (7.1) | - | 2 (4.4) |
| Other | 10 (32.3) | - | 3 (21.4) | - | 13 (28.9) |
| Missing | 1 (3.2) | - | 0 | - | 1 (2.2) |
| **CC-292** |  | **n = 27** | **n = 14** |  | **n = 41** |
| Median duration (range), days^a^ | - | 56.0 (12-542) | 35.5 (7-233) | - | 56.0 (7-542) |
| Median no. of cycles (range) | - | 2 (0-18) | 1 (0-8) | - | 2 (0-18) |
| Median relative dose intensity (range)^b^ | - | 1.0 (0.6-1.0) | 1.0 (0.6-1.0) | - | 1.0 (0.6-1.0) |
| ≥1 dose reduction, n (%) | - | 12 (44.4) | 4 (28.6) | - | 16 (39.0) |
| Adverse event | - | 5 (18.5) | 3 (21.4) | - | 8 (19.5) |
| Per protocol | - | 1 (3.7) | 1 (7.1) | - | 2 (4.9) |
| Other | - | 8 (29.6) | 1 (7.1) | - | 9 (22.0) |
| Missing | - | 1 (3.7) | 0 | - | 1 (2.4) |
| ≥1 dose interruption, n (%) | - | 14 (51.9) | 6 (42.9) | - | 20 (48.8) |
| Adverse event | - | 5 (18.5) | 3 (21.4) | - | 8 (19.5) |
| Per protocol | - | 1 (3.7) | 1 (7.1) | - | 2 (4.9) |
| Other | - | 8 (29.6) | 1 (7.1) | - | 9 (22.0) |
| Missing | - | 1 (3.7) | 0 | - | 1 (2.4) |
| **Rituximab** | **n = 19** | **n = 12** |  | **n = 30** | **n = 61** |
| Median duration (range), days^a^ | 112.0 (28-980) | 56.0 (28-784) | - | 56.0 (28-616) | 56.0 (28-980) |
| Median no. of cycles (range) | 4 (1-35) | 2 (1-28) | - | 2 (1-22) | 2 (1-35) |
| Median relative dose intensity (range)^b^ | 1.0 (0.9-1.1) | 1.0 (0.9-1.0) | - | 1.0 (0.5-1.0) | 1.0 (0.5-1.1) |
| ≥1 dose interruption, n (%) | 9 (47.4) | 2 (16.7) | - | 2 (6.7) | 13 (21.3) |
| Adverse event | 6 (31.6) | 2 (16.7) | - | 1 (3.3) | 9 (14.8) |
| Other | 5 (26.3) | 1 (8.3) | - | 2 (6.7) | 8 (13.1) |

Abbreviations: R, rituximab.

^a^Treatment duration defined as date of last derived dose – date of first dose + 1.

^b^Defined as the ratio of actual dose received to planned dose over the treatment period.

TABLE S3 Hematologic and gastrointestinal adverse events by treatment arm and dose level (safety population)

| **Arm A: Avadomide + CC-223 ± R** | **DL1 (n = 6)** | **DL2  (n = 6)** | **DL3  (n = 4)** | **DL4  (n = 6)** | **DL5 (n = 9)** |
| --- | --- | --- | --- | --- | --- |
| **Hematologic** |  |  |  |  |  |
| Neutropenia | 3 (50.0) | 4 (66.7) | 1 (25.0) | 2 (33.3) | 5 (55.6) |
| Anemia | 1 (16.7) | 3 (50.0) | 0 | 1 (16.7) | 4 (44.4) |
| Thrombocytopenia | 1 (16.7) | 4 (66.7) | 0 | 0 | 2 (22.2) |
| Febrile neutropenia | - | - | - | - | - |
| Leukopenia | - | - | - | - | - |
| **Gastrointestinal** |  |  |  |  |  |
| Diarrhea | 3 (50.0) | 4 (66.7) | 2 (50.0) | 5 (83.3) | 7 (77.8) |
| Abdominal | 2 (33.3) | 0 | 2 (50.0) | 2 (33.3) | 1 (11.1) |
| Nausea | 2 (33.3) | 0 | 0 | 1 (16.7) | 3 (33.3) |
| Vomiting | 2 (33.3) | 0 | 1 (25.0) | 1 (16.7) | 2 (22.2) |
| Constipation | 1 (16.7) | 0 | 1 (25.0) | 0 | 2 (22.2) |
| Dry mouth | 0 | 2 (33.3) | 1 (25.0) | 1 (16.7) | 0 |
| Stomatitis | 0 | 1 (16.7) | 0 | 1 (16.7) | 2 (22.2) |
| **Arm B: Avadomide + CC-292** | **DL1  (n = 6)** | **DL2  (n = 3)** | **DL3  (n = 6)** | **DL4  (n = 7)** | **DL5 (n = 5)** |
| **Hematologic** |  |  |  |  |  |
| Neutropenia | 3 (50.0) | 2 (66.7) | 1 (16.7) | 4 (57.1) | 2 (40.0) |
| Thrombocytopenia | 4 (66.7) | 3 (100) | 1 (16.7) | 1 (14.3) | 2 (40.0) |
| Anemia | 2 (33.3) | 1 (33.3) | 1 (16.7) | 0 | 2 (40.0) |
| Febrile neutropenia | 1 (16.7) | 1 (33.3) | 1 (16.7) | 1 (14.3) | 1 (20.0) |
| Leukopenia | 0 | 0 | 1 (16.7) | 1 (14.3) | 1 (20.0) |
| **Gastrointestinal** |  |  |  |  |  |
| Diarrhea | 5 (83.3) | 2 (66.7) | 1 (16.7) | 2 (28.6) | 2 (40.0) |
| Abdominal pain | 3 (50.0) | 1 (33.3) | 0 | 2 (28.6) | 2 (40.0) |
| Nausea | 2 (33.3) | 2 (66.7) | 2 (33.3) | 1 (14.3) | 1 (20.0) |
| Dyspepsia | 2 (33.3) | 1 (33.3) | 1 (16.7) | 0 | 0 |
| Vomiting | 3 (50.0) | 0 | 0 | 0 | 1 (20.0) |
| Constipation | 1 (16.7) | 0 | 1 (16.7) | 1 (14.3) | 0 |
| **Arm C CC-223 + CC-292** | **DL1  (n = 7)** | **DL2  (n = 7)** |  |  |  |
| **Hematologic** |  |  |  |  |  |
| Thrombocytopenia | 2 (28.6) | 3 (42.9) | - | - | - |
| Neutropenia | 1 (14.3) | 2 (28.6) | - | - | - |
| Anemia | 0 | 2 (28.6) | - | - | - |
| Leukopenia | 0 | 2 (28.6) | - | - | - |
| **Gastrointestinal** |  |  |  |  |  |
| Diarrhea | 4 (57.1) | 5 (71.4) | - | - | - |
| Dyspepsia | 2 (28.6) | 1 (14.3) | - | - | - |
| Nausea | 0 | 3 (42.9) | - | - | - |
| Vomiting | 2 (28.6) | 1 (14.3) | - | - | - |
| Dry mouth | 2 (28.6) | 0 | - | - | - |
| **Arm D: Avadomide + R** | **DL1  (n = 9)** | **DL2  (n = 3)** | **DL3  (n = 4)** | **DL4  (n = 8)** | **DL5 (n = 6)** |
| **Hematologic** |  |  |  |  |  |
| Neutropenia | 4 (44.4) | 1 (33.3) | 3 (75.0) | 5 (62.5) | 2 (33.3) |
| Anemia | 2 (22.2) | 0 | 1 (25.0) | 3 (37.5) | 2 (33.3) |
| Thrombocytopenia | 2 (22.2) | 0 | 0 | 2 (25.0) | 1 (16.7) |
| Febrile neutropenia | 1 (11.1) | 0 | 0 | 2 (25.0) | 0 |
| Leukopenia | 1 (11.1) | 0 | 0 | 2 (25.0) | 0 |
| Lymphopenia | 2 (22.2) | 0 | 1 (25.5) | 0 | 0 |
| **Gastrointestinal** |  |  |  |  |  |
| Constipation | 4 (44.4) | 0 | 0 | 0 | 2 (33.3) |
| Nausea | 2 (22.2) | 0 | 0 | 2 (25.0) | 1 (16.7) |
| Diarrhea | 1 (11.1) | 0 | 0 | 1 (12.5) | 1 (16.7) |
| Dysphagia | 1 (11.1) | 0 | 0 | 0 | 2 (33.3) |
| Vomiting | 2 (22.2) | 0 | 0 | 0 | 1 (16.7) |

Abbreviations: DL, dose level; R, rituximab.

TABLE S4 Treatment-related adverse events occurring in ≥10% of patients in either treatment arm
(safety population)

|  | **Arm A** | **Arm B** | **Arm C** | **Arm D** |
| --- | --- | --- | --- | --- |
|  | **Avadomide +  CC-223 ± R  (n = 31)** | **Avadomide + CC-292 ± R  (n = 27)** | **CC-292 +  CC-223  (n = 14)** | **Avadomide  + R  (n = 30)** |
| ≥1 TEAE, n (%) | 30 (96.8) | 24 (88.9) | 12 (85.7) | 24 (80.0) |
| **Hematologic** |  |  |  |  |
| Neutropenia | 15 (48.4) | 11 (40.7) | 2 (14.3) | 13 (43.3) |
| Anemia | 6 (19.4) | 6 (22.2) | - | 4 (13.3) |
| Thrombocytopenia | 6 (19.4) | 10 (37.0) | 4 (28.6) | - |
| Febrile neutropenia | - | 4 (14.8) | - | - |
| Leukopenia | - | 3 (11.1) | 2 (14.3) | 3 (10.0) |
| **Gastrointestinal** |  |  |  |  |
| Diarrhea | 19 (61.3) | 10 (37.0) | 7 (50.0) | 2 (6.7) |
| Nausea | 4 (12.9) | 6 (22.2) | 3 (21.4) | - |
| Vomiting | 5 (16.1) | 2 (7.4) | 2 (14.3) | - |
| Dyspepsia | - | 4 (14.8) | 2 (14.3) | - |
| **Other** |  |  |  |  |
| Hypokalemia | 5 (16.1) | - | - | - |
| Pyrexia | - | 3 (11.1) | - | - |
| Fatigue | 11 (35.5) | 6 (22.2) | - | 2 (6.7) |
| Rash | 8 (25.8) | 2 (7.4) | 2 (14.3) | 4 (13.3) |
| Rash maculopapular | 4 (12.9) | 2 (7.4) | - | 3 (10.0) |
| Decreased appetite | 7 (22.6) | - | - | 2 (6.7) |
| Hyperglycemia | 9 (29.0) | - | - | - |
| Proteinuria | 6 (19.4) | - | - | - |
| Pruritus | 3 (9.7) | 3 (11.1) | - | 5 (16.7) |
| Asthenia | - |  | 2 (14.3) | 4 (13.3) |
| Pneumonia | - | 4 (14.8) | - | - |
| Hyperbilirubinemia | - | 4 (14.8) | - | - |
| Dry skin | - | - | - | 3 (10.0) |
| AST increased | 3 (9.7) | 3 (11.1) | - | 2 (6.7) |
| ALT increased | 4 (12.9) | - | - | 3 (10.0) |

Abbreviations: ALT, alanine aminotransferase; AST, aspartate aminotransferase; R, rituximab;
TEAE, treatment-emergent adverse event.

TABLE S5 Treatment-emergent serious adverse events occurring in >1 patient by treatment arm (safety population)

|  | **Avadomide + CC-223 ± R  (n = 31)** | **Avadomide +  CC-292 ± R  (n = 27)** | **CC-292 + CC-223   (n = 14)** | **Avadomide + R  (n = 30)** |
| --- | --- | --- | --- | --- |
|  | **Arm A** | **Arm B** | **Arm C** | **Arm D** |
| ≥1 Any-grade SAE, n (%) | 19 (61.3) | 16 (59.3) | 8 (57.1) | 16 (53.3) |
| **Hematologic** | 2 (6.5) | 4 (14.8) | 1 (7.1) | 2 (6.7) |
| Febrile neutropenia | 0 | 4 (14.8) | 0 | 1 (3.3) |
| **Gastrointestinal** | 4 (12.9) | 1 (3.7) | 1 (7.1) | 4 (13.3) |
| **Infections** | 6 (19.4) | 3 (11.1) | 1 (7.1) | 6 (20.0) |
| Sepsis | 2 (6.5) | 1 (3.7) | 1 (7.1) | 0 |
| *Escherichia* pyelonephritis | 1 (3.2) | 0 | 0 | 0 |
| Pneumonia | 0 | 1 (3.7) | 0 | 2 (6.7) |
| **Neoplasms** | 2 (6.5) | 2 (7.4) | 0 | 2 (6.7) |
| **Other** |  |  |  |  |
| General health deterioration^a^ | 2 (6.5) | 6 (22.2) | 2 (14.3) | 3 (10.0) |
| Acute kidney injury | 2 (6.5) | 0 | 0 | 0 |
| Noncardiac chest pain | 0 | 2 (7.4) | 0 | 0 |
| Pyrexia | 0 | 2 (7.4) | 0 | 2 (6.7) |
| Asthenia | 1 (3.2) | 0 | 2 (14.3) | 1 (3.3) |
| Hypotension | 0 | 0 | 2 (14.3) | 0 |

Abbreviations: R, rituximab; SAE, serious adverse event.

^a^General health deterioration included clinical disease progression or worsening health status; patients did not meet criteria for radiographic disease progression.

TABLE S6 Dose-limiting toxicities (DLT population)

|  | **Arm A** | **Arm B** | **Arm C** | **Arm D** |
| --- | --- | --- | --- | --- |
|  | **Avadomide + CC-223 ± R  (n = 31)** | **Avadomide + CC-292 ± R  (n = 27)** | **CC-292 + CC-223   (n = 14)** | **Avadomide + R  (n = 30)** |
| Total DLT-evaluable, n | 26 | 24 | 10 | 24 |
| ≥1 DLT, n (%) | 4 (15.4) | 8 (33.3) | 4 (40.0) | 2 (8.3) |
| DL and DLT | | | | |
| DL1 | DLT-evaluable (n = 6)   - Grade 3 febrile neutropenia (n = 1) | DLT-evaluable (n = 6)   - Grade 3 febrile neutropenia, increased hepatic enzyme, and hyperbilirubinemia and grade 4 hyperamylasemia and hyperlipasemia (n = 1) - Grade 4 thrombocytopenia  (n = 1) - Grade 4 increased lipase  (n = 1) | DLT-evaluable (n = 6)   - Grade 4 confusional state and depressed level of consciousness (n = 1) - Grade 3 urinary tract infection (n = 1) | DLT-evaluable (n = 5)   - Grade 3 febrile neutropenia and thrombocytopenia  (n = 1) |
| DL2 | DLT-evaluable (n = 4)   - Grade 3 rash (n = 1) - Grade 3 thrombocytopenia, maculopapular rash, hyperglycemia, and mucosal inflammation (n = 1) | DLT-evaluable (n = 3)   - Grade 3 febrile neutropenia^a^ (n = 1) - Grade 4 neutropenia (n = 1) | DLT-evaluable (n = 4)   - Grade 4 neutropenia, grade 3 thrombocytopenia, and grade 1 erythematous rash (n = 1) - Grade 4 thrombocytopenia (n = 1) | DLT-evaluable (n = 3)  – |
| DL3 | DLT-evaluable (n = 3)  – | DLT-evaluable (n = 4)  – | NA | DLT-evaluable (n = 4)  – |
| DL4 | DLT-evaluable (n = 6)  – | DLT-evaluable (n = 6)   - Grade 2 abdominal pain and fatigue (n = 1) | NA | DLT-evaluable (n = 6)   - Grade 4 neutropenia  (n = 1) |
| DL5 | DLT-evaluable (n = 7)   - Grade 4 hypercalcemia  (n = 1) | DLT-evaluable (n = 5)   - Grade 3 hepatotoxicity (n = 1) - Grade 3 myalgia (n = 1) | NA | DLT-evaluable (n = 6)  – |

Abbreviations: DL, dose level; DLT, dose-limiting toxicity; NA, not applicable; R, rituximab.

^a^Resulted in death.

**TABLE S7** Efficacy by dose cohort

| **Arm A: Avadomide +  CC-223 ± R** | **Avadomide 2 mg QD + CC-223 20 mg QD**  **(n = 6)** | **Avadomide 2 mg QD + CC-223 30 mg QD**  **(n = 6)** | **Avadomide 2 mg QD +  CC-223 20 mg QD + R**  **(n = 4)** | **Avadomide 3 mg QD 5/7 d + CC-223 20 mg QD + R**  **(n = 6)** | **Avadomide 3 mg QD 5/7 d + CC-223 20 mg QD + R**  **(n = 9)** |
| --- | --- | --- | --- | --- | --- |
| ORR, n (%) [95% CI] | 1 (16.7) [0.4-64.1] | 0 | 1 (25.0)  [0.6-80.6] | 2 (66.7)  [22.3-95.7] | 3 (33.3)  [7.5-70.1] |
| CR | 0 | 0 | 1 (25.0) | 2 (33.3) | 1 (11.1) |
| PR | 1 (16.7) | 0 | 0 | 2 (33.3) | 2 (22.2) |
| SD | 2 (33.3) | 1 (16.7) | 0 | 1 (16.7) | 4 (44.4) |
| PD | 3 (50.0) | 3 (50.0) | 3 (75.0) | 1 (16.7) | 2 (22.2) |
| Missing | 0 | 2 (33.3) | 0 | 0 | 0 |
| **Arm B:**  **Avadomide + CC-292 ± R** | **Avadomide 1 mg QD + CC-292 500 mg BID**  **(n = 6)** | **Avadomide 2 mg QD + CC-292 500 mg BID**  **(n = 3)** | **Avadomide 1 mg QD 5/7 d + CC-292 500 mg BID**  **(n = 6)** | **Avadomide 1 mg QD 5/7 d + CC-292 500 mg BID + R**  **(n = 7)** | **Avadomide 2 mg QD 5/7 d + CC-292 500 mg BID + R**  **(n = 5)** |
| ORR, n (%) [95% CI] | 1 (16.7)  [0.4-64.1] | 1 (33.3)  [0.8-90.6] | 0 | 2 (28.6)  [3.7-71.0] | 3 (60.0)  [14.7-94.7] |
| CR | 1 (16.7) | 1 (33.3) | 0 | 0 | 1 (20.0) |
| PR | 0 | 0 | 0 | 2 (28.6) | 2 (40.0) |
| SD | 2 (33.3) | 0 | 3 (50.0) | 2 (28.6) | 0 |
| PD | 2 (33.3) | 1 (33.3) | 1 (16.7) | 3 (42.9) | 2 (40.0) |
| Missing | 1 (16.7) | 1 (33.3) | 2 (33.3) | 0 | 0 |
| **Arm C:**  **CC-223 + CC-292** | **CC-122 15 mg QD + CC‑292 500 mg BID**  **(n = 7)** | **CC-122 20 mg QD + CC‑292 500 mg BID**  **(n = 7)** |  |  |  |
| ORR, n (%) [95% CI] | 0 | 0 |  |  |  |
| CR | 0 | 0 |  |  |  |
| PR | 0 | 0 |  |  |  |
| SD | 3 (42.9) | 3 (42.9) |  |  |  |
| PD | 3 (42.9) | 2 (28.6) |  |  |  |
| Missing | 1 (14.3) | 2 (28.6) |  |  |  |
| **Arm D:**  **Avadomide + R** | **Avadomide 2 mg QD + R (n = 9)** | **Avadomide  2 mg QD 5/7 d + R  (n = 3)** | **Avadomide  3 mg QD 5/7 d + R (n = 4)** | **Avadomide  4 mg QD 5/7 d + R (n = 8)** | **Avadomide  3 mg QD 5/7 d + R (n = 6)** |
| ORR, n (%) [95% CI] | 1 (11.1)  [0.3-48.2] | 2 (66.7)  [9.4-99.2] | 1 (25.0)  [0.6-80.6] | 2 (25.0)  [3.2-65.1] | 1 (16.7)  [0.4-64.1] |
| CR | 1 (11.1) | 1 (33.3) | 0 | 0 | 0 |
| PR | 0 | 1 (33.3) | 1 (25.0) | 2 (25.0) | 1 (16.7) |
| SD | 2 (22.2) | 0 | 0 | 0 | 0 |
| PD | 4 (44.4) | 1 (33.3) | 2 (50.0) | 5 (62.5) | 4 (66.7) |
| Missing | 2 (22.2) | 0 | 1 (25.0) | 1 (12.5) | 1 (16.7) |

Abbreviations: CR, complete response; DL, dose level; ORR, objective response rate; PD, progressive disease; PR, partial response; R, rituximab; SD, stable disease.

FIGURE S1 Change in the percentage of apoptotic cells over time (A) and *in vivo* tumor growth response after combination treatment with avadomide and rituximab (B). Natural killer cell activity against DOHH or RL FL cell lines determined by flow cytometry is shown (A). Female CB17 severe combined immunodeficiency mice implanted subcutaneously with DOHH2 cells were administered vehicle and avadomide once daily and rituximab given 1 mg/kg once weekly (B). SEM, standard error of the mean; TGI, tumor growth inhibition. mm^3^

FIGURE S2 CONSORT diagram. Diagram of patient flow and disposition as well as analysis populations (safety, efficacy, pharmacokinetic, and biomarker). All screened patients (N = 158) had DLBCL. *One patient who did not meet inclusion criteria (≥1 site of measurable disease) was enrolled in error. ^†^Avadomide intermittent schedule (5/7 d); all other dosing regimens were QD. ^‡^Noncompliance with study drug. ^§^Patient started a prohibited medication. ^||^Patient achieved a complete response and underwent transplant. 5/7 d, 5 consecutive days out of 7; AE, adverse event; DL, dose level; PD, pharmacodynamics; PK, pharmacokinetics; R, rituximab.

FIGURE S3 Summary of pharmacokinetic parameters and mean plasma concentration-time profiles of (A) CC-223 and (B) CC-292. Summary of pharmacokinetic parameters and mean plasma concentration-time profiles of (A) CC-223 and (B) CC-292. AUC_0-24_, area under the plasma concentration-time curve from time 0 to 24 hours; AUC_0-∞_, area under the plasma concentration-time curve from time 0 to infinity; CL/F, apparent clearance; C_max_, maximum plasma drug concentration; t_1/2,_ terminal half-life; T_max_, time to C_max_; Vz/F, apparent volume of distribution during the terminal phase. Data are geometric means (CV%) except for T_max_, for which medians (min, max) are presented.


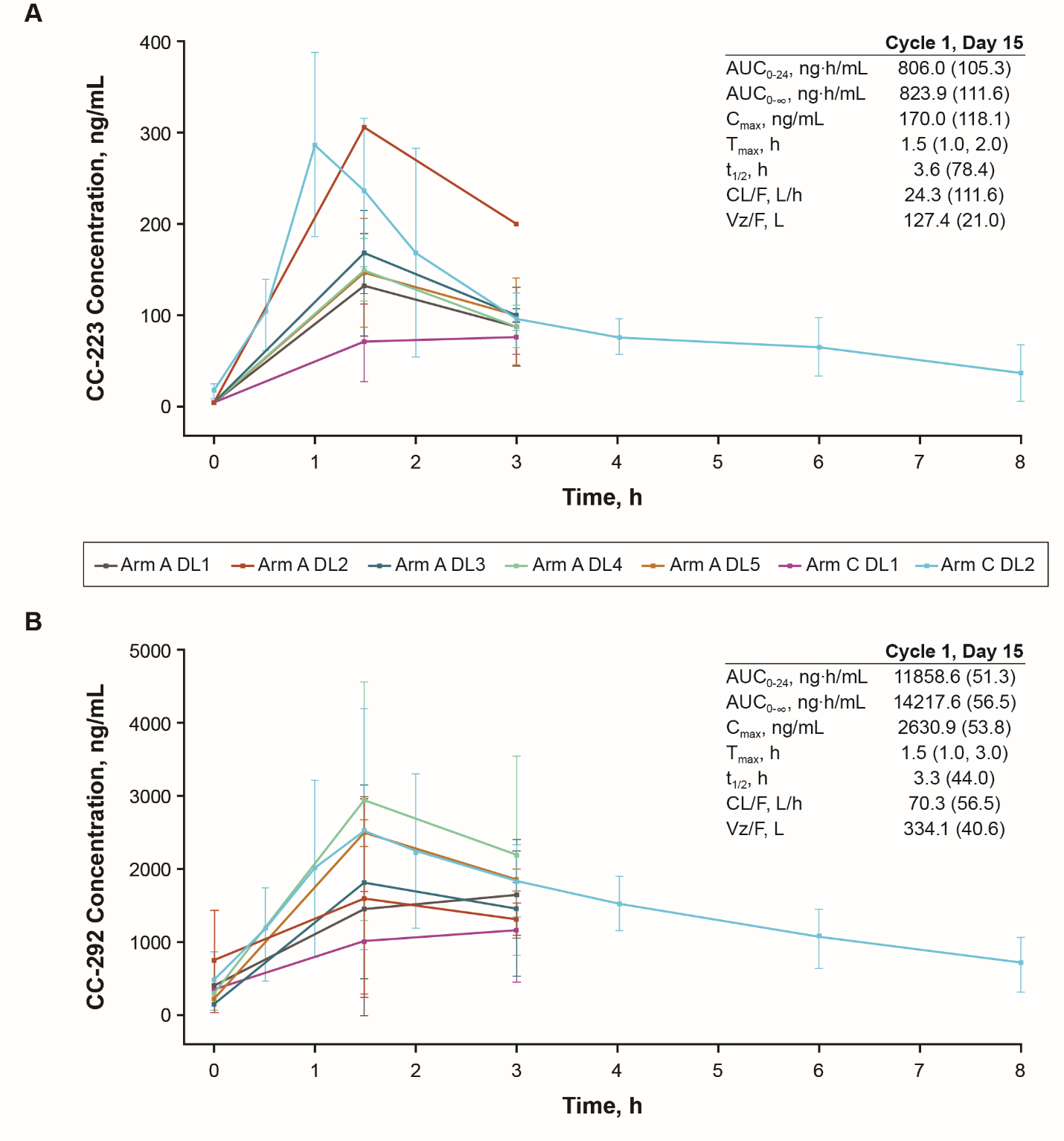

Supplement: Supplementary file 1 — SUPPORTING INFORMATION [file JHA2-3-139-s001.docx]
